# Genomes of the “*Candidatus* Actinomarinales” Order: Highly Streamlined Marine Epipelagic Actinobacteria

**DOI:** 10.1128/mSystems.01041-20

**Published:** 2020-12-15

**Authors:** Mario López-Pérez, Jose M. Haro-Moreno, Jaime Iranzo, Francisco Rodriguez-Valera

**Affiliations:** a Evolutionary Genomics Group, División de Microbiología, Universidad Miguel Hernández, San Juan, Alicante, Spain; b Centro de Biotecnología y Genómica de Plantas, Universidad Politécnica de Madrid (UPM) – Instituto Nacional de Investigación y Tecnología Agraria y Alimentaria (INIA), Madrid, Spain; c Institute for Biocomputation and Physics of Complex Systems (BIFI), University of Zaragoza, Zaragoza, Spain; d Research Center for Molecular Mechanisms of Aging and Age-related Diseases, Moscow Institute of Physics and Technology, Dolgoprudny, Russia; Scripps Institution of Oceanography

**Keywords:** Actinomarinales, single-amplified genomes, marine *Actinobacteria*, streamlined genomes

## Abstract

“*Candidatus* Actinomarinales” was defined as a subclass of exclusively marine *Actinobacteria* with small cells and genomes. We have collected all the available genomes in databases to assess the diversity included in this group and analyzed it by comparative genomics. We have found the equivalent of five genera and 18 genomospecies. They have genome reduction parameters equal to those of freshwater actinobacterial “*Candidatus* Nanopelagicales” or marine alphaproteobacterial *Pelagibacterales*. Genome recruitment shows that they are found only in the photic zone and mainly in surface waters, with only one genus that is found preferentially at or below the deep chlorophyll maximum. “*Ca*. Actinomarinales” show a highly conserved core genome (80% of the gene families conserved for the whole order) with a saturation of genomic diversity of the flexible genome at the genomospecies level. We found only a flexible genomic island preserved throughout the order; it is related to the sugar decoration of the envelope and uses several tRNAs as hot spots to increase its genomic diversity. Populations had a discrete level of sequence diversity similar to other marine microbes but drastically different from the much higher levels found for *Pelagibacterales*. Genomic analysis suggests that they are all aerobic photoheterotrophs with one type 1 rhodopsin and a heliorhodopsin. Like other actinobacteria, they possess the F420 coenzyme biosynthesis pathway, and its lower reduction potential could provide access to an increased range of redox chemical transformations. Last, sequence analysis revealed the first “*Ca*. Actinomarinales” phages, including a prophage, with metaviromic islands related to sialic acid cleavage.

**IMPORTANCE** Microbiology is in a new age in which sequence databases are primary sources of information about many microbes. However, in-depth analysis of environmental genomes thus retrieved is essential to substantiate the new knowledge. Here, we study 182 genomes belonging to the only known exclusively marine pelagic group of the phylum *Actinobacteria*. The aquatic branch of this phylum is largely known from environmental sequencing studies (single-amplified genomes [SAGs] and metagenome-assembled genomes [MAGs]), and we have collected and analyzed the available information present in databases about the “*Ca.* Actinomarinales.” They are among the most streamlined microbes to live in the epipelagic zone of the ocean, and their study is critical to obtain a proper view of the diversity of *Actinobacteria* and their role in aquatic ecosystems.

## Abstract (precis)

Microbiology is in a new age in which sequence databases are primary sources of information about many microbes. However, in-depth analysis of environmental genomes thus retrieved is essential to substantiate the new knowledge.

## INTRODUCTION

One major drawback of ocean productivity is biomass density. In the absence of buoyancy mechanisms, cells are heavier than seawater and sink. That creates a fundamental problem of recycling since light barely penetrates beyond the upper 200 m. Hence, marine ecosystems are often nutrient limited due to the loss of inorganic nutrients (N, P, Fe, and others) to the dark ocean. As an added problem, warm water is lighter than cold water and creates thermal stratification across the water column that blocks the exchange of nutrients between the photic zone and the deeper strata. Consequently, aquatic environments, particularly deep and warm basins, tend to be highly oligotrophic. To import nutrients under such conditions, prokaryotic cells have to be small, with cell volumes that often are under 0.01 μm^3^ (as a reference Escherichia coli cells are about 1 μm^3^) (1, 2). Such reduced volume implies restriction in the amount of DNA that a cell can carry, limiting the size of their genomes.

Studies of the ocean microbiome have revealed that it is dominated by small bacteria adapted to survive in oligotrophic conditions with a significant reduction in genome size, few pseudogenes, short intergenic spacers, and low GC content, i.e., they have streamlined genomes, such as members of the alphaproteobacterial *Pelagibacterales* (SAR11 clade) and the cyanobacterial *Prochlorococcaceae* (3). The above-mentioned microbes serve as models for size and genome reduction, but there are many other examples of aquatic microbes with similar characteristics. In freshwater, the sublineage of SAR11, LD12 (4), or the most abundant clade of *Actinobacteria*, the “*Candidatus* Nanopelagicales,” have very small genome sizes (ca. 1.2 Mb) (5). In the ocean, we find many other examples such as the marine ammonia‐oxidizing thaumarchaeon “*Candidatus* Nitrosopelagicus brevis” (6), the recently described groups of heterotrophic marine thaumarchaea abundant in mesopelagic waters (7), the methylotrophic *Betaproteobacteria* of clade OM43 (8), and the “*Ca.* Actinomarinales” (9).

The reduced genome and cell sizes of some “*Ca.* Actinomarinales” and their worldwide distribution have been known for some years (9, 10). They are the only known exclusively marine, free-living, planktonic *Actinobacteria* (9) since the other, distantly related, marine pelagic *Actinobacteria*, the *Acidimicrobiales* (11), are also found abundantly in freshwater lakes. To our knowledge, the only report of “*Ca.* Actinomarinales” outside the global ocean was in the South Basin of the brackish Caspian Sea (ca. 1.5% salinity) (12). In a previous work (9), a single composite genome was assembled from a collection of metagenomic fosmids from the Mediterranean deep chlorophyll maximum (DCM) (9). The 16S rRNA could be found among the fosmids and was affiliated with the formerly described marine *Actinobacteria* clade (13). The cells were characterized by fluorescent *in situ* hybridization (FISH) and flow cytometry and described as the smallest free-living bacteria (9). The admittedly chimeric genome reconstructed from the fosmids indicated a very small size, and it had very small intergenic spacers and also very low GC content (the lowest value found for any actinobacterium) (9). The subclass “*Candidatus* Actinomarinidae,” order “*Candidatus* Actinomarinales,” suborder “*Candidatus* Actinomarineae,” family “*Candidatus* Actinomarinaceae,” and species “*Candidatus* Actinomarina minuta” were proposed to accommodate this single reconstructed genome (9). However, because of its chimeric features, it was not included in the Genome Taxonomy Database (GTDB) (14). Presently, the corresponding class in GTDB is *Acidimicrobiia*. Until 2019, only a few genomes assembled from metagenomes (MAGs) had been included within this clade (15, 16). However, a large collection of single-amplified genomes (SAGs) from the tropical and subtropical euphotic ocean has been recently released providing insights into the heterogeneity and genomic composition of the marine microbiome (17). Among them, almost 200 new genomes were classified as “*Candidatus* Actinomarina.”

We have conducted a phylogenomic analysis indicating the presence of five different genera (average nucleotide identity [ANI] of <70%). Clustering closely related genomes allowed us to reconstruct the first complete genomes and, in this way, conduct comparative genomic analysis. The results provide information into the genomic makeup, ecogenomics, microdiversity, and evolutionary dynamics of this diverse group of microbes. On the basis of these analyses, we propose the establishment of new genera plus a more accurate description of the previously proposed “*Ca.* Actinomarina.”

## RESULTS

### Phylogenomics of the order “*Ca.* Actinomarinales.”

To collect as much genomic diversity as possible and perform a phylogenomic classification of the whole clade, we collected all genomes corresponding to the class *Acidimicrobiia* according to the GTDB (*Actinobacteria* based on the NCBI classification) as well as several reference genomes from nearby classes (*Actinobacteria*, *Coriobacteriia*, and *Themoleophilia*) that have several representatives from marine and freshwater ecosystems. After removing those that did not pass the established quality criteria (>50% completeness and <5% contamination), a total of 1,814 genomes were used to perform a phylogenomic tree using 262 shared genes (see Fig. S1A in the supplemental material). In the end, 182 genomes (largely SAGs, together with seven MAGs [15, 16]) clustered in the same branch with “*Ca*. Actinomarina minuta,” classified within the order TMED189 in the GTDB nomenclature or “*Ca.* Actinomarinales” by the NCBI (see Table S1 in the supplemental material).

10.1128/mSystems.01041-20.1 FIG S1 (A) Phylogenomic tree with complete genomes of the class *Acidimicrobiia* according to the GTDB (*Actinobacteria* based on the NCBI classification). A total of 262 concatenated conserved proteins were used to generate a maximum likelihood tree. The genomes of nearby classes *Actinobacteria*, *Coriobacteriia*, and *Themoleophilia* were used as outgroup. The branches have been colored according to the order to which they belong. The reference genome of “*Ca*. Actinomarina minuta” is marked with a star and highlighted in red. (B) Phylogenetic positioning of “*Ca.* Actinomarinales” genomes based on 16S rRNA genes using genomes of the class *Acidimicrobiia* from panel A as an outgroup. Branches of the tree and names were colored according to the genera as follows: G1 (green), G2 (blue), G3 (brown), G4 (purple,) and G5 (red). An asterisk indicates the positioning of the first composite genome “*Ca*. Actinomarina minuta” derived from combining fosmid clones. Download FIG S1, PDF file, 0.2 MB. Copyright © 2020 López-Pérez et al. 2020 López-Pérez et al. This content is distributed under the terms of the Creative Commons Attribution 4.0 International license.

10.1128/mSystems.01041-20.8 TABLE S1 Detailed information about the genomes used in this study. Download Table S1, XLSX file, 0.02 MB. Copyright © 2020 López-Pérez et al. 2020 López-Pérez et al. This content is distributed under the terms of the Creative Commons Attribution 4.0 International license.

**FIG 1:**
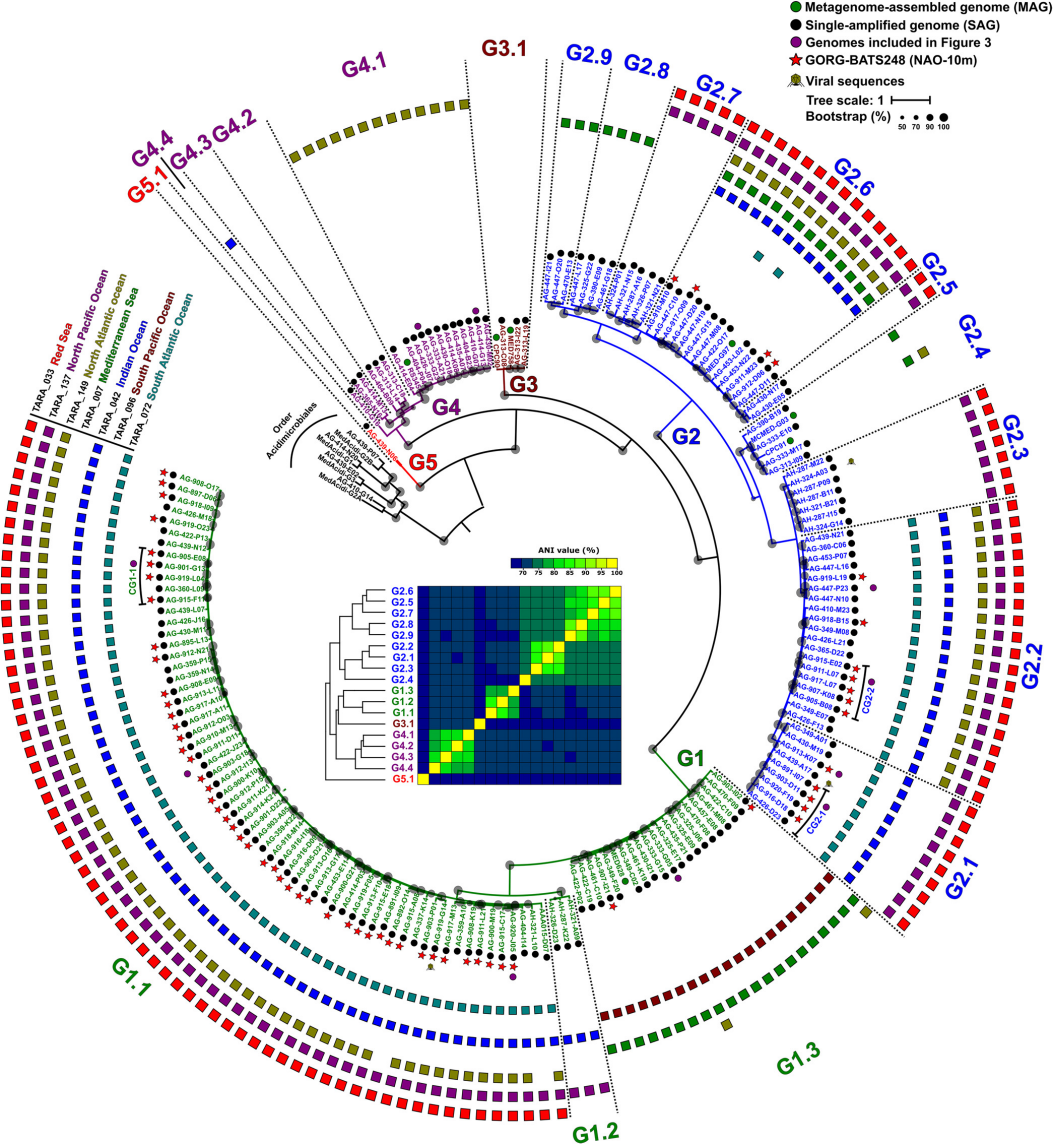
Maximum likelihood phylogenomic tree of the “*Ca.* Actinomarinales” genomes. Colored dots next to the genome identifier indicate the origin of the genome that is MAG (green) or SAG (black). Red stars show genomes from a single sample collected at the BATS station. Purple dots indicate genomes used to produce the composite genomes and genomes used in Fig. 3. Branches of the tree and names were colored according to the genera as follows: G1 (green), G2 (blue), G3 (brown), G4 (purple), and G5 (red). Dotted lines divide the different genomospecies within each genus. Outer rings show the presence (RPKGs >5) in surface TARA-reference metagenomes covering different geographical sites. Bootstrap values are indicated as black circles on the nodes. The inset shows the average nucleotide identity (ANI) matrix of genomospecies colored by genera.

To compare and cluster the “*Ca.* Actinomarinales” genomes, we conducted a second phylogenomic analysis using genomes of the sister order *Acidimicrobiales* as an outgroup. Most genomes were clustered in four main branches, which seemed to represent putative genera based on ANI values of ca. 70%, named G1 to G4 (Fig. 1). A single SAG appeared as an outgroup (AG-439-N06, labeled G5 in Fig. 1). Most of the genomes of G1 come from a single sample collected at the Bermuda Atlantic Time-series Study (BATS) station (17) (highlighted with a red star in Fig. 1). To assign the first composite genome “*Ca.* Actinomarina minuta” derived from combining fosmid clones (9), we constructed a 16S rRNA gene tree (Fig. S1B), confirming the phylogenomic groups and identifying the 16S rRNA gene of the “*Ca.* Actinomarina” (9) within G2, for which we propose to retain this genus denomination.

### Genomospecies and ecological distribution patterns.

Next, we sought to delve into the distribution patterns of each genome using a broad set of metagenomic data sets (Materials and Methods). Before metagenome read recruitment, the rRNA ribosomal operon was removed from the genomes (18). To consider presence in a metagenomic sample positive, we established a minimum threshold of five reads per kilobase of genome and gigabase of metagenome (RPKG) and genome coverage of >70% with an identity threshold of ≥98% (Table S2). Interestingly, we found that within each genus there were groups of genomes with similar patterns of recruitment and RPKG values, which clustered together in the phylogenomic tree (Fig. 1 and Table S2). The similarity within these groups was >90% ANI. These ecogenomic clusters were considered genomospecies (18). For simplicity, the mean recruitment of all the genomes within each genomospecies was used to estimate their distribution.

10.1128/mSystems.01041-20.9 TABLE S2 Recruitment values (RPKG) for all the genomes at 98% identity in *Tara* Oceans metagenomes (A), GEOTRACES cruise (B), BATS and HOT stations seasonal variations (C), Red Sea vertical profile (D), North Pacific Ocean, HOTs, vertical profile (E), and Western Mediterranean Sea vertical profile (F). Download Table S2, XLSX file, 1.5 MB. Copyright © 2020 López-Pérez et al. 2020 López-Pérez et al. This content is distributed under the terms of the Creative Commons Attribution 4.0 International license.

**FIG 2:**
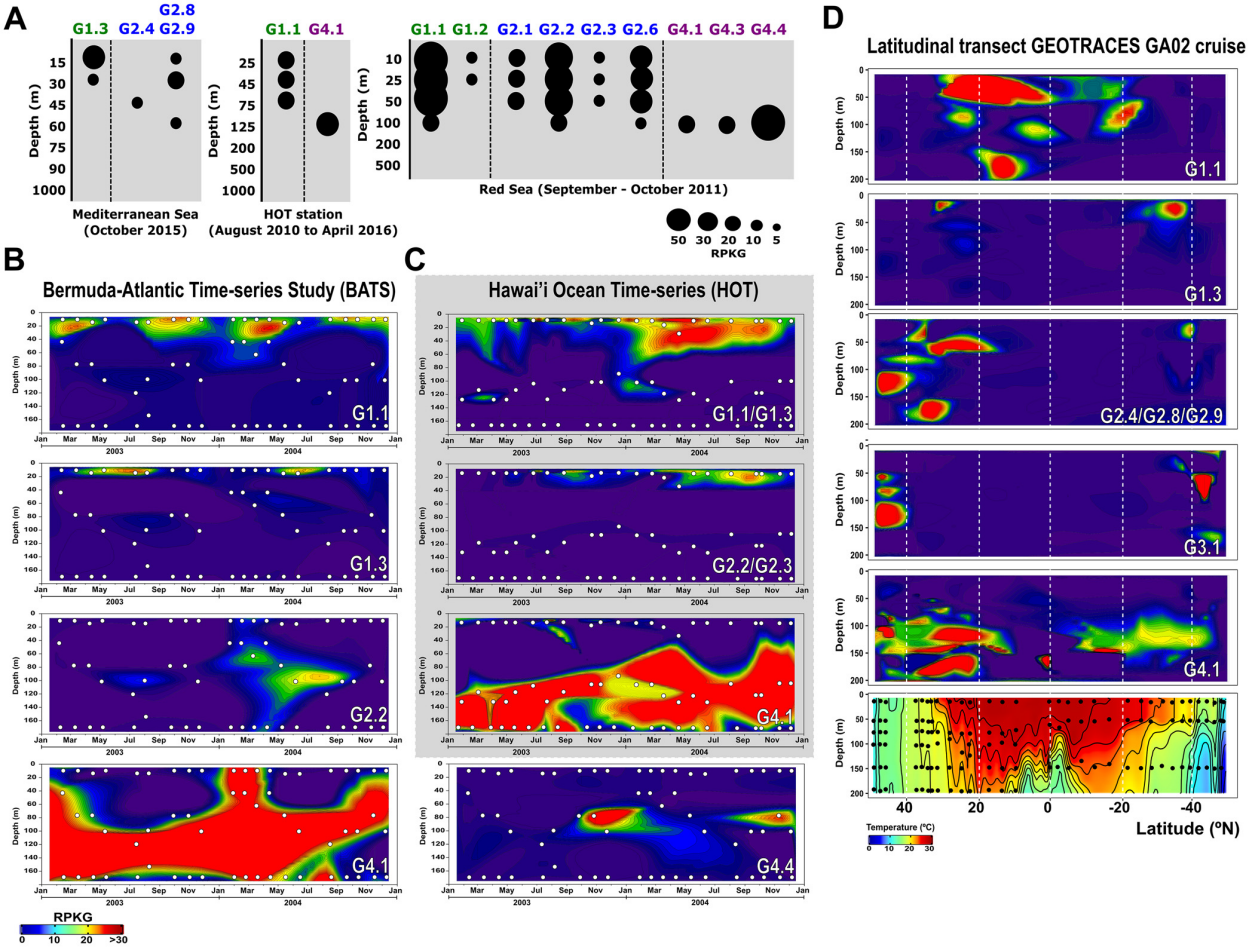
Relative abundance of “*Ca*. Actinomarinales” genomospecies in: (A) vertical profiles from Western Mediterranean Sea, Red Sea, and North Pacific Ocean; (B) 2-year metagenomic time series collected at monthly intervals at BATS; and (C) HOT stations during the GEOTRACES cruises. (D) Latitudinal transect following the GEOTRACES GA02 cruise. The temperature profile is shown in the last box. White and black dots indicate locations of the metagenomic samples.

Among the *Tara* Oceans metagenomic data sets, some genomospecies were more abundant in specific regions such as the Mediterranean Sea and the Atlantic North East (G1.3, G2.4, G2.8, and G2.9) or the Pacific South West (G2.7), while others showed a more global distribution (G1.1, G2.1, G2.2, and G2.6) (Fig. 1 and Fig. S2). None appeared in the Southern Ocean or in meso- and bathypelagic samples, indicating a clear association to the photic zone and warmer waters. G5, with only one representative, was not present in any of the samples analyzed. However, given the lack of time series or depth profiles in this worldwide data set, the meaning of the differential recruitment along these transects is hard to judge. For this reason, we used smaller data sets to detect clear ecological patterns in vertical, latitudinal, and seasonal variations. Vertical profiles across the photic zone in different geographical points, Western Mediterranean Sea (16), Red Sea (19), and North Pacific Ocean (20), showed that most genomospecies have a predilection for the upper layers of the epipelagic zone (the upper 20 m) (Fig. 2A). This was also the case for some *Pelagibacterales* genomospecies (18). However, like the *Pelagibacterales* genomospecies Ia.3/VIII (18), genomospecies within “*Ca.* Actinomarinales” G4 showed a clear preference for the deeper photic zone (DCM, 50- to 100-m depth).

10.1128/mSystems.01041-20.2 FIG S2 Relative abundance of “*Ca.* Actinomarinales” genomospecies in *Tara* Ocean metagenomes. Stations were ordered according to the depth indicated to the left. At the top, the bar indicates the number of stations where each genospecies is present (RPKG > 5). Download FIG S2, PDF file, 0.03 MB. Copyright © 2020 López-Pérez et al. 2020 López-Pérez et al. This content is distributed under the terms of the Creative Commons Attribution 4.0 International license.

In the 2-year time-series metagenomic data from BATS stations (21), which is seasonally stratified, two genomospecies alternated on the surface. Thus, G1.1 was more abundant in the cooler months, when the water column mixes and nutrients up-well near the surface, while G1.3 was much more prevalent in the warm stratified surface (Fig. 2B). However, the most abundant genomospecies was the deeper dweller G4.1 that was restricted to the DCM (50- to 100-m depth), or below, in the stratified season, but appeared across the whole mixed water column between January and May (Fig. 2B). The other G4 genomospecies (G4.4) present at this location was limited to layers at 60 to 80 m during the mixed period (Fig. 2B). In the permanently stratified Hawaii Ocean Time series (HOT) station, these G4 genomospecies only appeared below 60 m, while G1 and G2 genomospecies appeared near the extremely oligotrophic surface (Fig. 2C).

We also found differences in the latitudinal gradients from North to South Atlantic Ocean (GEOTRACES GA02 cruise [21]). Along this gradient, G1.1 was more abundant in tropical samples, while G1.3 and G2 were more prevalent in temperate zones. However, G3.1 was present only at higher latitudes, above 40°N or S. This preference for colder waters may be partially responsible for a lower representation of these genomes in collections of SAGs, since most samples used to retrieve them come from tropical and subtropical areas.

### “*Ca.* Actinomarinales” genome reduction.

A comparison of some general features of the largest genomes belonging to each genus is shown in Table 1. We have also included in the comparison a representative of the marine *Pelagibacterales* (HTCC7211), Prochlorococcus marinus (MED4), the actinobacterial freshwater clade “*Ca*. Nanopelagicales” acI (IMCC25003), and the well-known copiotroph E. coli K-12. The values indicate that “*Ca.* Actinomarinales” with an overall GC content of ca. 32.5% and 2 bp as median intergenic distance have genomes more streamlined than “*Ca.* Nanopelagicales” and P. marinus and more similar to those of the *Pelagibacterales*. It is remarkable the small number of paralog genes (nearly half of those found for the *Pelagibacterales* representative), particularly considering that a paralog NADH dehydrogenase (*nuo*) cluster with 11 genes was present (see below). The small number of sigma factors, the total absence of identifiable two-component systems, mobile genetic elements, toxin-antitoxin, or CRISPR systems, and the increase in the average size of operons, are other characteristics shared with the *Pelagibacterales*. We found only three insertion sequence elements, one of them associated with a restriction-modification system and another two located in the genome AG-891-I09 (genomospecies G1.1) inserted in one of the tRNAs at the boundary of the cell wall biosynthesis and modifications, flexible genomic island 1 (fGI1) (see below).

**TABLE 1:** Genomic features of the largest genomes belonging to each “*Ca.* Actinomarinales” genus versus reference genomes^*a*^

Genome (group)	Type	Genome size (bp)	GC content (%)	No. of proteins	No. of proteins/Mb	Avg gene size (bp)	Coding density (%)	Median intergenic spacer (bp)	No. of paralogs (no. of paralogs/100 proteins)	Avg no. of genes/operon	No. of sigma factors
AG-915-F11 (G1)	SAG^*b*^	1,104,260	32.4	1,182	1,070	900.0	97	2	44 (3.7)	4.6	2
AG-913-K07 (G2)	SAG^*b*^	1,121,776	32.7	1,194	1,064	903.6	97	3	39 (3.3)	4.8	2
AG-313-C08 (G3)	SAG	902,589	33.9	973	1,078	889.5	97	3	29 (3.0)	4.7	2
AG-414-G13 (G4)	SAG^*b*^	1,110,800	31.9	1,184	1,066	900.3	97	3	48 (4.1)	4.7	2
HTCC7211 (*Pelagibacterales*)	Isolate	1,456,888	29.0	1,547	1,062	907.5	97	3	93 (6.0)	4.7	2
IMCC25003 (“*Ca.* Nanopelagicales”)	Isolate	1,353,947	49.1	1,360	1,004	926.6	96	11	60 (4.4)	4.4	3
*P. marinus* MED4	Isolate	1,657,990	30.8	1,916	1,156	765.6	90	38	130 (6.8)	3.0	5
E. coli K-12	Isolate	4,641,652	50.8	4,316	930	940.5	88	68	650 (15.1)	3.4	7

a Reference genomes are indicated by gray shading.

b Complete SAG, estimated by genome alignment.

### Comparative genomic analysis.

**FIG 3:**
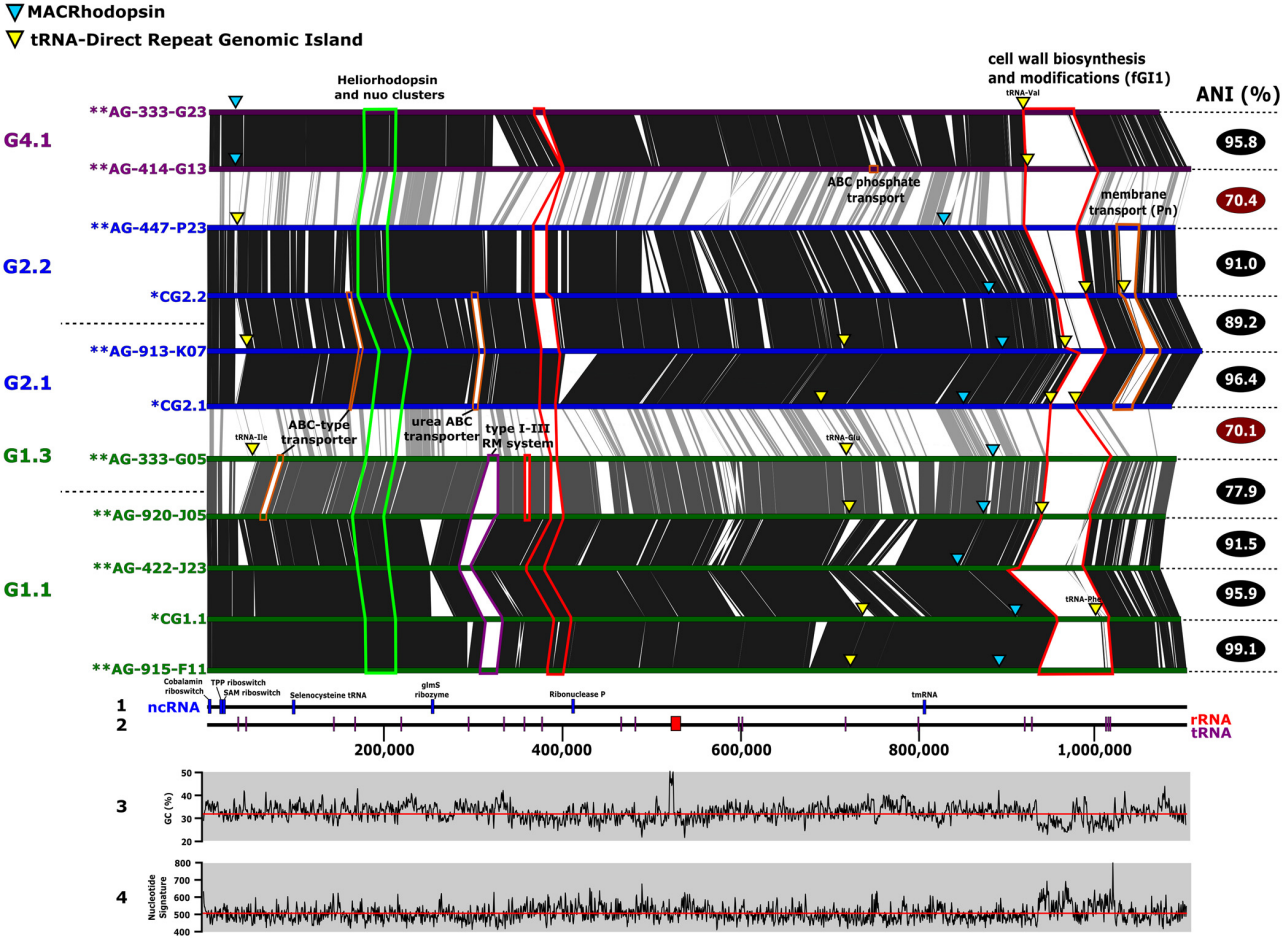
Whole-genome alignment of the most complete and composite genomes of “*Ca.* Actinomarinales”. Genomes have been linearized and rearranged to start at the *dnaA* gene. Synteny and sequence similarity are indicated by vertical lines connecting the genomes. Flexible genomic islands (fGI) have been highlighted in different colors. fGI for genes involved in cell wall biosynthesis and modifications (marked in red), genes related to membrane transport (marked in orange), and genes coding for a restriction-modification system I-III (marked in purple) are highlighted. The region containing the heliorhodopsin gene and two *nuo* clusters are marked in green. Blue and yellow triangles indicate the presence of the MACRhodopsin and tRNA-direct repeat genomic islands, respectively. At the bottom of the figure, panel 1 shows the location of noncoding RNA (ncRNA), panel 2 shows the locations of tRNA and rRNA genes, panel 3 shows GC content, and panel 4 shows nucleotide signatures.

The absence of pure culture reference genomes hampers genomic comparisons, so to assess synteny and analyze the variable regions, multiple SAGs were coassembled to obtain composite reference genomes (Fig. S3) (see Materials and Methods), one for each of the genomospecies G1.1, G2.1, and G2.2. The size of these composite genomes was ca. 1.1 Mb, larger than previously predicted (9), but still among the smallest genomes of free-living cells described so far (Table 1), even compared to the marine *Pelagibacterales* whose average size is 1.3 Mb (22). We found only one copy of the 16S, 23S, and 5S rRNA ribosomal genes forming a single operon that is located right after the terminus determined by the GC skew (data not shown). To increase the number of genomes to compare and based on the approximate size of the composite genomes (1.1 Mb), we also included another eight SAGs with that approximate size, since they were likely nearly complete. In the end, 11 genomes were used for comparison, belonging to five genomospecies and three genera (G1, G2, and G4). Despite the divergence among the different genera (ANI of ca. 70%), synteny was well preserved (Fig. 3).

10.1128/mSystems.01041-20.3 FIG S3 Reconstruction of composite genomes (CGs) belonging to genomospecies G1.1, G2.1, and G2.3. A nucleotide comparison of five highly related SAGs (ANI > 99%) is shown. Contigs are colored according to the SAG sequence. Download FIG S3, PDF file, 0.03 MB. Copyright © 2020 López-Pérez et al. 2020 López-Pérez et al. This content is distributed under the terms of the Creative Commons Attribution 4.0 International license.

One flexible genomic island (fGI1) (23), with equivalent location and different gene content, even within the same species, was present across the whole order. Not surprisingly, this fGI appears to be involved in cell envelope polysaccharide biosynthesis (glycotype [24]). This has already been reported for most bacteria and archaea. It seems to be a universal feature that, within individual species, many different combinations of genes coding for components involved in the biosynthesis of the outermost layer of the cell are found for different strains. Incidentally, similar fGIs have been described in the freshwater actinobacteria “*Ca.* Nanopelagicales” (5). This glycotype island is present in all the genomes of the order at the same relative location, on the left replichore, and relatively close to the replication origin (Fig. 3). The conserved location allowed us to recover 43 complete islands in different SAGs. Their genes were clustered (95% identity) and functionally annotated through the KEGG database (Table S3). Gene family’s annotation and metabolic pathways related to “Glycosyltransferases.” “Lipopolysaccharide biosynthesis” or “Amino sugar and nucleotide sugar metabolism” clearly indicated that these genes are involved in the synthesis of a structural polysaccharide or capsule. At this point, the structure and composition of the cell wall of aquatic (low-GC) actinobacteria (class *Acidimicrobiia* by the GTDB taxonomy) are not known. No evidence of genes involved in the synthesis of mycolic acids could be found, so the presence of an outer membrane in these actinobacteria seems unlikely. Complete fGI1 sizes range from 43 to 79 kb and, as is usually the case for this type of fGI, have different genomic parameters with lower GC content (28%) (25). A former study of fGIs coding for glycotypes in other bacteria and archaea indicated that their extraordinary level of diversity derives from their frequent exchange, by double crossover recombination, that allows the complete replacement by other gene clusters, coming from different strains or species (or even genera) (23, 26). However, in “*Ca.* Actinomarinales,” there seems to be a different mode of gene swapping with partial replacement by smaller gene cassettes (Fig. S4A), as previously shown for additive fGIs in other microbes (23, 24). The five tRNAs within each fGI1 act as targets for the insertion of these gene cassettes (Fig. S4A). Although the most common position is at both ends of the island, i.e., the island expands from the ends, they sometimes suffer from rearrangements that place them closer to the center (Fig. S4A). The most recent insertions are still identifiable by the tell-tale direct repeat of part of the tRNA gene at the end of the inserted cassette (highlighted as red arrows in Fig. S4A). This dynamic situation is well represented by the case of the G2.2 genomes (AG-447-P23 and AG-447-N10) which have an ANI of >98%. Both genomes have similar gene contents on the island but have acquired small fragments that start to differentiate their fGI1 into different versions (Fig. S4B). We have also found evidence of gene cassette transfer between different genera, specifically AG-435-A07 (G4.1) and AG-919-G14 (G1.1) (Fig. S4A). However, in this case, the exchange must be older since the similarity between the genes has decreased to ca. 75%. We screened the genomes for more tRNA fragment direct repeats indicative of other hot spots for site-directed recombination. We found two other loci connected to tRNAs (Ile and Glu, highlighted as yellow triangles in Fig. 3); next to them, there were genes related to ABC transporters. It should be noted that in the cases of tRNAs Asp, Ile and Phe, they were found to be single copies in the genome, and thus, any damage to their sequence could be lethal. The alignment of the genomes did not allow for the detection of any other fGIs found in all the genera, but G2 and G4 both had another fGI, at different locations in each genus but at a conserved position within each (Fig. 3). They contained a restriction-modification system type I-III and transporters, including a phosphonate one, respectively. Although they are hyperdiversified regions, their genomic features are similar to those of the core (GC content, intergenic distance, and nucleotide signature) (Fig. 3).

10.1128/mSystems.01041-20.4 FIG S4 (A) Schematic representation of the fGI1 gene cassettes found in different strains representatives of “*Ca.* Actinomarinales.” Red and black arrows above the genomes indicate the 3′ end of the tRNA gene section that is duplicated and represent a hallmark of an integration event and the presence of the tRNA, respectively. (B) Comparison of the fGI1 involved cell envelope polysaccharide biosynthesis found in two highly similar genomes of the genomospecies G2.2 (ANI 98.3%). Purple and black arrows above the genomes indicate the 3′ end of the tRNA gene section that is duplicated and represent a hallmark of an integration event and the presence of the tRNA, respectively. Synteny and sequence similarity are indicated by vertical lines connecting the genomes. The insertion events that separate the two closely related islands are highlighted in yellow. Download FIG S4, PDF file, 0.1 MB. Copyright © 2020 López-Pérez et al. 2020 López-Pérez et al. This content is distributed under the terms of the Creative Commons Attribution 4.0 International license.

10.1128/mSystems.01041-20.10 TABLE S3 KEGG annotation of gene families contained in the fGI1. Download Table S3, XLSX file, 0.02 MB. Copyright © 2020 López-Pérez et al. 2020 López-Pérez et al. This content is distributed under the terms of the Creative Commons Attribution 4.0 International license.

**FIG 4:**
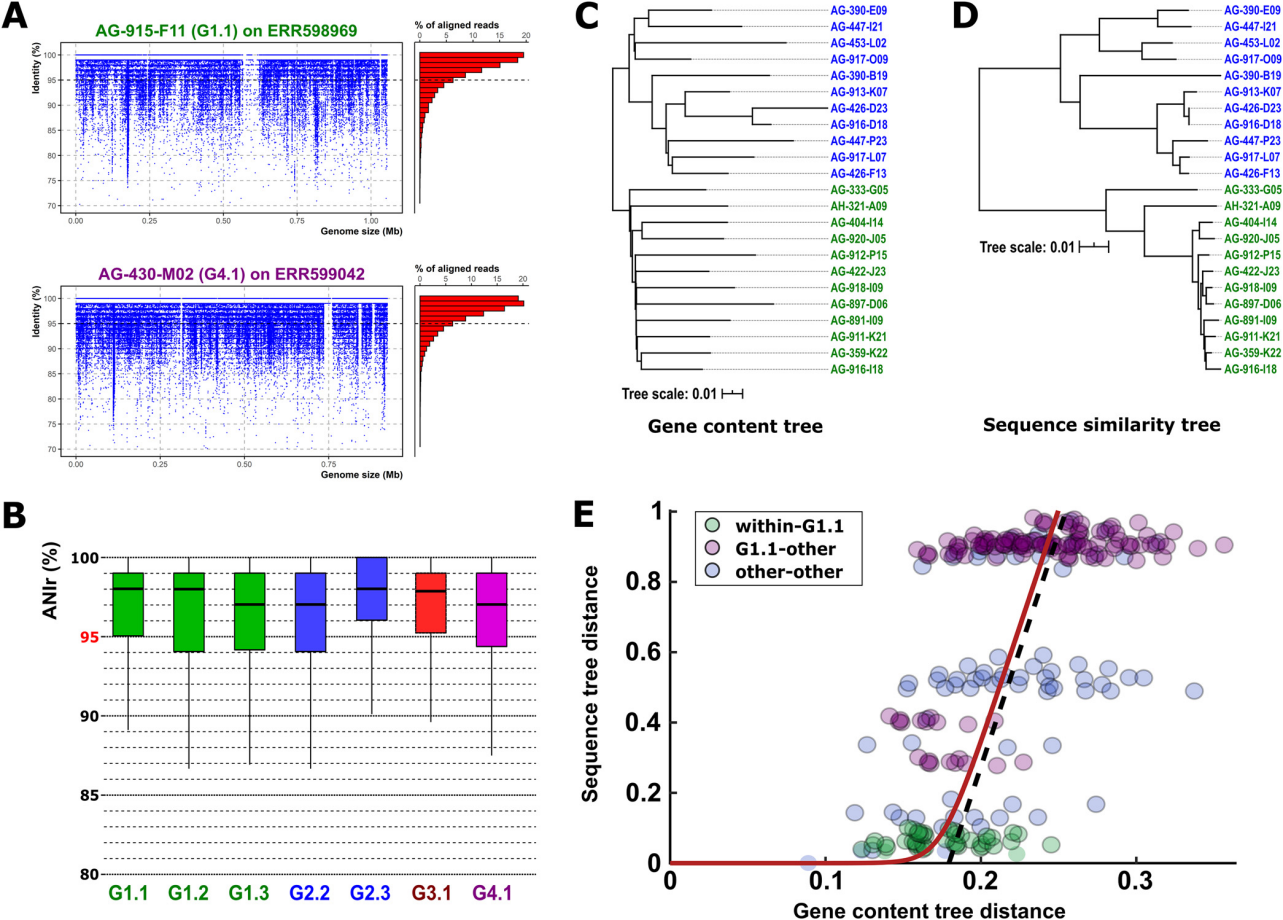
(A) Linear recruitment plot of the reference genomes AG-915-F11 (G1.1) and AG-430-M02 (G4.1). Each blue dot represents a metagenomic read. The histogram on the right shows the relative percentage of aligned reads in intervals of 1% identity. The black dashed line indicates the species threshold (95%). (B) Boxplot indicating the average nucleotide identity based on metagenomic reads (ANIr). (C and D) Phylogenetic trees based on the concatenated alignment of genes shared by all members of the group (C) and proportional number of estimated gene gain and loss events (D). (E) Comparison of gene content divergence and core-gene sequence divergence in “*Ca.* Actinomarinales” genomes. Each circle represents a pair of genomes; colors indicate pairs of genomes within the G1.1 genomospecies (green), between the G1.1 and a different genomospecies (purple), and between members of genomospecies other than G1.1 (blue). The red line shows the best fit to the recombination-driven delay model of genome evolution. The dashed line provides a visual clue highlighting the transition from the recombination-bound regime to the linear divergence regime.

We analyzed the intrapopulation sequence diversity within each group using an average nucleotide identity calculated by metagenomic reads (ANIr). Linear recruitment allowed us to differentiate between those genomospecies that were represented in the sample (Fig. 4A and Fig. S5A) and those in which a close relative was the most abundant (Fig. S5B). Using the values of three genomes within each genomospecies in three metagenomes, we found that the whole class is made up of discrete populations with a lower intrapopulation sequence diversity (ANIr, ca. 97%) than the species threshold (95%) (Fig. 4B). In contrast, *Pelagibacterales* populations are characterized by ANIr values below 95%, and the threshold in the linear recruitment plots is located above 80% identity, suggesting much higher intrapopulation diversity (27).

10.1128/mSystems.01041-20.5 FIG S5 Linear recruitment plot of genomes that are present in the metagenomic sample (A) and not present in the metagenomic sample (B). Each blue dot represents a metagenomic read. The histogram on the right shows the relative percentage of aligned reads in intervals of 1% identity. The black dashed line indicates the species threshold (95%). Download FIG S5, PDF file, 0.7 MB. Copyright © 2020 López-Pérez et al. 2020 López-Pérez et al. This content is distributed under the terms of the Creative Commons Attribution 4.0 International license.

### Pangenome evolution.

Although missing parts of the genome in SAGs can alter some analysis, in this study we have used the pangenome as a defining unit for each genomospecies to assess the patterns of genomic variation. Only those genomospecies with at least five genomes were considered. In the end, nine genomospecies were included in the analysis (G1.1, G1.3, G.2.1, G2.2, G2.3, G2.6, G2.7, G3.1, and G4.1) containing a total of 4,932 gene family clusters (70% identity). The number of clusters shared by all was 932, which we consider the core of the order “*Ca.* Actinomarinales.” This is a very large figure for genomes of ca. 1.1 Mb (complete genomes had an average number of proteins of 1,160) and separated by such sequence divergence (ca. 70% ANI). These results show an exceptionally large proportion of shared genes (ca. 80%) across the whole “*Ca.* Actinomarinales” order. To put these values into perspective, we applied the same analysis to five *Pelagibacterales* genomospecies within subclade 1a.3 (18). In this case, we obtained 663 gene families shared by all of the genomes, which represents ca. 50% of the average genome, similar to what has been found previously for the SAR11 clade (22). In the case of “*Ca.* Nanopelagicales,” in 13 genomes of “*Candidatus* Planktophila” (5), 852 genes (59 to 68% of the genome) made up the core.

To analyze this phenomenon in more detail and cast light on the mechanisms underlying gene and genome evolution, we investigated the relationship between the evolution of the core genome (sequence-level divergence) and the loss and gain of genes through transfer (gene content divergence). Specifically, we compared the leaf-to-leaf distances in the sequence similarity tree, built from the concatenated alignment of strict single-copy core genes (Fig. 4D), with those from the gene content tree, whose branch lengths are proportional to the expected number of gene gains and losses experienced by a lineage (Fig. 4C). We found a very strong delay in core-gene sequence divergence for the flexible gene content divergence (Fig. 4E). Qualitatively similar delays have been observed in other groups of bacteria, and their origin has been attributed to the homogenizing action of intrapopulation homologous recombination on the sequences of core genes (28–30). Notably, the delay of approximately one unreported substitution per site estimated for “*Ca.* Actinomarinales” lies at the top end of the range observed in bacteria (28). Such a large delay suggests that homologous recombination plays a fundamental role in keeping genomospecies of “*Ca*. Actinomarina” (e.g., within G1.1) genetically cohesive while maintaining high rates of gene turnover (green circles in Fig. 4E). In contrast, genomes from distinct genomospecies have reached a regime of linear divergence, in which both gene and genome evolution proceed in parallel (blue and purple circles in Fig. 4E). The relative rate of gene turnover versus substitutions in such a linear regime is unusually low (approximately 0.08 compared to typical values of the order of 0.5 in other marine bacteria with larger genomes, such as *Alteromonas* and *Shewanella* [28]). The combination of a large delay in core-gene sequence evolution and a low gene turnover rate in the linear regime is compatible with the observation that most of the variability in the accessory genome of “*Ca*. Actinomarina” is concentrated at or near the terminal branches of the phylogenetic tree (Fig. 4C), that is, within the genomospecies level.

### Metabolism and lifestyle clues.

**FIG 5:**
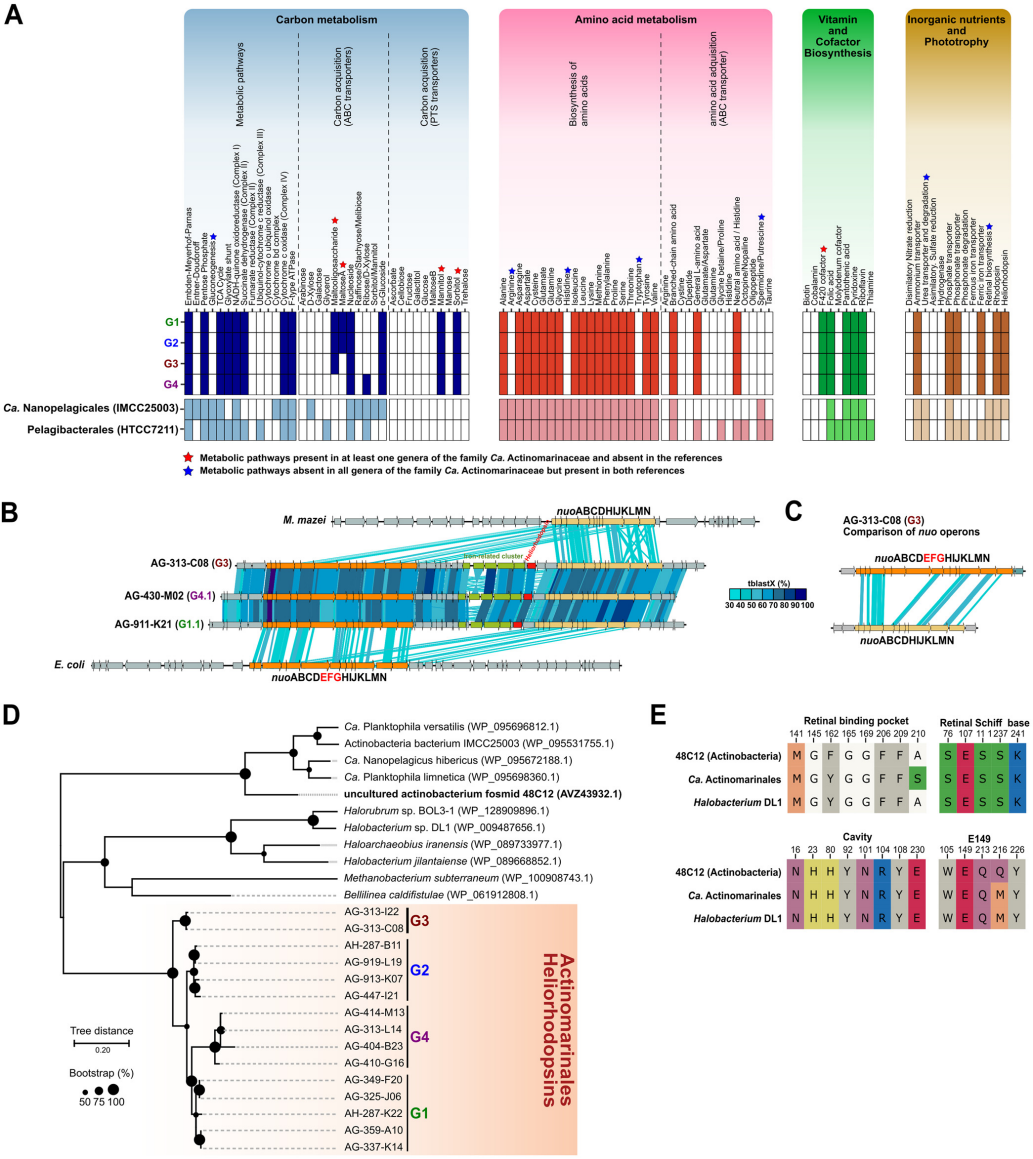
(A) Inferred metabolism of the four “*Ca.* Actinomarinales” genera based on the KEGG database. “*Ca.* Nanopelagicales” (IMCC25003) and *Pelagibacterales* (HTCC7211) were added for the comparison against other streamlined genomes. (B) Genomic alignment (in amino acids) of the two *nuo* paralogs close to the heliorhodopsin. *nuo* operons of Escherichia coli and Methanosarcina mazei were added for the comparison. (C) Alignment (in amino acids) between the two *nuo* paralogs detected in “*Ca.* Actinomarinales”, indicating that the subunits EFG were missing in one copy. (D) Maximum likelihood phylogenetic tree of the heliorhodopsin protein. Accession numbers for the reference heliorhodopsins are indicated within parentheses. Bootstrap values are indicated as black circles on the nodes. (E) Conserved key residues among the reference 48C12, retrieved from a fosmid of an uncultured freshwater *Actinobacteria* (GenBank accession number MF737519), *Halobacterium* DL1, and “*Ca*. Actinomarinales” heliorhodopsins.

The initial incomplete “*Ca*. Actinomarina minuta” genome was considered a photoheterotroph (9), and our data extend this hypothesis for the whole “*Ca.* Actinomarinales” order, although due to its incompleteness, we have excluded from the analysis the only representative of G5. Again, the high homogeneity of the group is evident at this level since there are hardly any differences at the predicted metabolic pathways among the groups. For this reason, the results have been compared with the reference genomes shown in Table 1. Results indicated that members of G1 to G4 encode components involved in glycolysis via the Embden-Meyerhof-Parnas pathway and components involved in the pentose phosphate pathways, but not the Entner-Doudoroff pathway (Fig. 5A). All of them can continue the oxidation of organic matter to CO_2_ by the tricarboxylic acid (TCA) cycle. Complex III of the electron transport chain was missing. They all shared most of the detected ABC and phosphotransferase system (PTS) transporters, encoding for components involved in the uptake and degradation of nucleosides, α-glucosides, mannitol, and sorbitol (Fig. 5A). Only members of G4, mainly found at the DCM or deeper waters (see above) encode components involved in the transport of ribose/d-xylose, while the degradation of maltose and maltooligosaccharides seems to be characteristic of surface “*Ca.* Actinomarinales”. The low number of transporters for carbohydrates seems to be a common trait among small genomes, such as “*Ca.* Nanopelagicales” and *Pelagibacterales*. However, it is remarkable that compared with *Pelagibacterales*, “*Ca.* Actinomarinales” genes encode PTS transporters. This difference in the transport of sugars could provide “*Ca.* Actinomarinales” an edge not to be completely outcompeted by *Pelagibacterales* in the same oligotrophic marine surface waters.

A major difference with the other streamlined microbes of Fig. 5A and Table 1 is that all “*Ca.* Actinomarinales” genomes were consistently auxotrophic for arginine, histidine, and tryptophan, indicating a dependence for these amino acids, that must be taken from the environment. Some “*Ca.* Nanopelagicales” are also auxotrophic for certain amino acids, but the degree of auxotrophy is variable depending on the strain (5). In the same way as with auxotrophy, there does not seem to be an equal distribution in the retinal synthesis within the order. This was attributed to differential losses in a genome reduction process (25). In the case of “*Ca.* Actinomarinales,” these auxotrophies must come from a distant common ancestor, and different species do not seem to be undergoing independent genome reduction at this level. Overall, “*Ca.* Actinomarinales” seem to prefer exogenous amino acids as carbon sources. In this sense, their genomes encoded several copies of branched-chain amino acid transporters, as well as for general l-amino acid and neutral amino acid/histidine transporters. Besides, the presence of a cyanophycinase in their genomes indicated that they could degrade the cyanophycine copolymer of arginine and aspartic acid produced by cyanobacteria, so they could easily incorporate arginine into their diet. The pattern of vitamin dependence is similar to the other streamlined microbes, such as “*Ca.* Nanopelagicales” (5) and *Pelagibacterales* (31) (Fig. 5A). It is remarkable that “*Ca.* Actinomarinales”, conversely to the freshwater “*Ca.* Nanopelagicales,” can synthesize the F420 cofactor, and several genes encoding F420-dependent oxidoreductases have been detected in their genomes. “*Ca.* Actinomarinales” genes encoded transporters for the uptake of ferric ions, ammonium, phosphate, and phosphonates, although no genes involved in the degradation of phosphonates were detected in any of the genomes (Fig. 5A).

### Structure and diversity of rhodopsins in “*Ca.* Actinomarinales.”

All the streamlined genomes, including “*Ca.* Actinomarinales,” encoded proton-pumping rhodopsins, while only “*Ca.* Actinomarinales” and “*Ca.* Nanopelagicales” encoded a heliorhodopsin, whose function remains unclear (32). However, we found that “*Ca.* Actinomarinales,” conversely to the other photoheterotrophs analyzed here, did not harbor the genes involved in the synthesis of the retinal chromophore (Fig. 5A).

The completeness of several of the “*Ca.* Actinomarinales” genomes analyzed allowed us to identify the presence of the two types of rhodopsins (type 1 and type 3) in most of their genomes. The heliorhodopsin gene was always found at the same location, between two copies of the complex I NADH dehydrogenase (*nuo*) operon (Fig. 5B). Between the *nuo* operons, in addition to the heliorhodopsin gene, we identified a cluster related to iron acquisition coding for an Fe^3+^ ABC transporter, a ferrochelatase, and an iron-dependent repressor. Interestingly, one of the *nuo* paralog clusters was truncated, missing the subunits EFG (Fig. 5C), which are involved in NADH oxidation to NAD^+^ (33). This truncated cluster is similar (up to 35% amino acid identity) to the cluster found in the euryarchaeon Methanosarcina mazei (34) (Fig. 5B) that is known to couple complex I of the respiratory chain with the F420 cofactor (34). The phylogenetic tree placed the “*Ca.* Actinomarinales” heliorhodopsin equidistant to the haloarchaeal ones and other actinobacterial homologs (Fig. 5D). However, the study of key residues identified in the structure of the reference heliorhodopsin 48C12 (32, 35) showed that they may share the same, yet unknown, function (32, 35) (Fig. 5E).

In “*Ca.* Actinomarinales,” the type 1 rhodopsin was already described (9) and named marine actinobacterial clade (MACRhodopsin [MACR]). More recently, a fosmid from the Red Sea belonging to the same group displayed proton-pumping activity (36), and the three-dimensional (3D) structure was established (37). The sequences of the MACR cluster together and close to proteorhodopsins (Fig. S6B) but are more distant to the other actinobacterial rhodopsins such as acidirhodopsins (11) or actinorhodopsins (38). The analysis of key amino acids (data not shown) also indicates that they are all green-light-absorbing outward proton pumps.

10.1128/mSystems.01041-20.6 FIG S6 (A) Genome alignment (in amino acids) of the type 1 rhodopsin gene neighbors among contigs from three “*Ca.* Actinomarinales” SAGs, AG-911-K21 (genomospecies G1.1), AG-313-C08 (G3), and AG-430-M02 (G4.1). We included in the comparison two fosmids: MedDCM-OCT-S36-C22, representing the reference MACRhodopsin of “*Ca.* Actinomarina minuta” (GenBank accession number KC811130) and the first fosmid (uncultured bacterium EIL26B11) where the light-driven proton pump function of these rhodopsins was demonstrated (accession number KT201089). The figure shows the genomic rearrangement of rhodopsin, photolyase, NAD transhydrogenase, and a hypothetical protein within genomospecies G4.1. (B) Maximum likelihood phylogenetic tree of type 1 rhodopsins. Between brackets the accession number is indicated. Bootstrap values are indicated as black circles on the nodes. Download FIG S6, PDF file, 0.1 MB. Copyright © 2020 López-Pérez et al. 2020 López-Pérez et al. This content is distributed under the terms of the Creative Commons Attribution 4.0 International license.

In contrast to heliorhodopsin, the MACR gene was found at two different loci along the genome depending on the genus (Fig. 3). Three genera (G1 to G3) had the MACR gene located next to the photolyase (likely cotranscribed) along the right replichore. However, in G4, the MACR gene appeared on the left replichore, next to the photolyase but in the opposite strand and with a relatively large intergenic spacer (Fig. S6A). On the boundaries of the insertion, a small (84-amino-acid) hypothetical membrane protein was duplicated, although the identity between the two paralogs was only 57%. Intriguingly, the closest relatives to these proteins affiliated with *Bacteroidetes* and *Firmicutes* with low but significant identities (48%). This duplication was found only in G4; the other genera had only a single copy (Fig. S6A). It is also interesting that the G4 MACR gene is the most divergent among all the genera (Fig. S6B). G4 genomospecies seem specialized in deeper waters (see above) what might explain these differences in their relationship to light.

### First bona fide *Actinobacteria* phages and prophage.

One of the advantages of single-cell sequencing is the ability to obtain sequences of viruses that are inside or attached to the bacterial cells and therefore infer the host (39). Genome annotation and analysis revealed three sequences containing virus-related genes, including major capsid proteins, the large subunit of phage terminases, portal proteins, phage tail proteins as well as actinobacterium-specific transcription factor WhiB (Fig. S7A) (40). The absence of terminal repetitions did not allow us to know whether they are complete sequences. The similarity among the three sequences was very low in addition to the fact that the hosts belonged to different genomospecies (G1.1, G2.1, and G2.3). Except for AH-324-A03, which was barely detected in any station, metagenomic read recruitment in *Tara* Oceans virome samples of the other two viral sequences followed distribution patterns similar to their hosts and was undetectable in the Southern Ocean and deep water viromes (Fig. S7B). The longest sequence was located associated with the G1.1 genome AG-919-G14 (29 kb and 35.5% GC). The size of the auxiliary metabolic gene coding for WhiB is twice that of the bacterial homolog. It appears that this gene has been fused with another gene that has a domain related to an ADP-ribosyltransferase toxin. This toxin was also found in freshwater phages infecting the acI lineage of *Actinobacteria*, and a protective role against eukaryotic predators was suggested (40). Based on linear metagenomic recruitment, the variable part is formed by several proteins involved in the removal of sialic acid from the cell wall to allow the virus access to host receptors (Fig. S7C). Another viral sequence, with a size of 27 kb (33.6% GC), was found in the G2.1 genomospecies SAG AG-439-A17. Interestingly, the last corresponded to a 24-kb (36.5% GC) prophage inserted in the tRNA-Val in the genome of AH-324-A03 (G2.3) (Fig. S7A). Although previous efforts using metagenomics have revealed putative phages of these hosts (41, 42), these are the first bona fide “*Ca.* Actinomarinales” phages. However, as in *Pelagibacterales* (43), the presence of prophages seems to be rare in these microbes.

10.1128/mSystems.01041-20.7 FIG S7 “*Ca.* Actinomarinales” viral sequences. (A) Virus-related sequences found in “*Ca.* Actinomarinales” SAGs. Genes colored in green and blue indicate viral or bacterial origin, respectively. (B) Recruitments of the viral sequences within the different metagenomic data sets of the Tara Oceans virome. The left axis and dotted line indicate depth of sample. (C) Linear recruitment plot of the sequences from the stations marked as a star in panel B. Download FIG S7, PDF file, 0.4 MB. Copyright © 2020 López-Pérez et al. 2020 López-Pérez et al. This content is distributed under the terms of the Creative Commons Attribution 4.0 International license.

## DISCUSSION

Actinobacteria are now recognized as major players of aquatic habitat communities (44). Members of the actinobacterial order “*Ca.* Nanopelagicales” are actually among the most abundant and frequent components of freshwater microbiomes (45). The order “*Ca.* Actinomarinales” is closer to the *Acidimicrobiales* but is only found in marine epipelagic waters (9). They represent the most streamlined genomes of the phylum *Actinobacteria* as could be expected from microbes specialized in such nutrient-limited environments that require high surface-to-volume ratios. They are nonmotile and have little regulatory capabilities. All these properties are shared with *Pelagibacterales* and fit with the lifestyle of pelagic oligotrophs that dominate the microbiome in the upper layers of the epipelagic ocean (2). The “*Ca.* Actinomarinales” overlap largely with the *Pelagibacterales* in terms of habitat with similar widespread distribution (46). However, they always represent a much smaller fraction of the community. Their distant freshwater relatives of the *Acidimicrobiales* or the “*Ca.* Nanopelagicales” appear to be at that level much more successful and better competitors of the freshwater *Pelagibacterales*-like Fonsibacter (4). It is remarkable how most of the genomospecies analyzed here prefer the near-surface (upper 20 m) waters, despite their rather harsh conditions (high UV light intensity, nutrient depletion, and variable conditions due to the hydrodynamic action of wind and waves [47]). But the same is largely true also of many *Pelagibacterales* species. However, in both cases, there are taxa (“*Ca.* Actinomarinales” G4, *Pelagibacterales* Ia.3/VIII genomospecies, and subclade IIb [46]) that dwell at deeper levels, at or below the DCM depths, that offer a much more stable and protected habitat.

The genomes analyzed do not permit us to glimpse how the survival strategy of “*Ca.* Actinomarinales” is different from their competitors like some of the *Pelagibacterales*. They are both photoheterotrophs, although aquatic actinobacteria also have a heliorhodopsin gene. It is hard to venture a role for heliorhodopsins, they are certainly not proton pumps, and a possible function as an enzyme reducing either carbonate or nitrate has been proposed (35). In any case, the role must be important for the survival of the microbe on account of its conservation. It could also be a key to the success of both freshwater and marine actinobacteria that seem to have this combination of proton pump and heliorhodopsin as a constant in their genomes. Like other actinobacteria (48), “*Ca.* Actinomarinales” genomes, despite their limited size, have two large *nuo* clusters, one that has the features required to transfer electrons from NADH to the respiratory chain and another that might use coenzyme F420 instead. This coenzyme is widespread in this phylum (49), but its biological role is unclear (50). However, its low redox potential (it can accept electrons from much weaker donors) might facilitate the degradation of some resilient compounds (as shown for soil actinobacteria) (51). Regardless, considering their widespread presence in the epipelagic ocean and their overall diversity, the “*Ca.* Actinomarinales” represent an important player in the microbial ecology of the oligotrophic ocean that should be further studied to understand their role in such a key ecosystem.

One recent discovery about at least some *Pelagibacterales* species is that they are present in nature in populations with low ANIr (ca. 92%), i.e., they have high intrapopulation diversity that among other reasons could be attributed to very high levels of recombination (intra- and interspecies) (27). This does not seem to hold for “*Ca.* Actinomarinales” species that are made up of discrete populations with an ANIr of ca. 97%, similar to other nonstreamlined genomes (27), suggesting fewer concurrent species or less interspecies recombination. Another major difference at the level of comparative population genomics between these two streamlined microbes is the size of the core genome, ca. 80% of the genes are shared by all the genera of “*Ca.* Actinomarinales” described here, a figure that is barely 50% among similar diversity ranges in the *Pelagibacterales*. Moreover, the flexible genome diversity of the first seems to reach saturation at the level of the genomospecies (Fig. 4E). Both observations point toward an evolutionary scenario where strong selective pressure on genome size is combined with great genomic plasticity: given a particular gene, it is either essential and maintained throughout the lineage (thus the conservation of the core and its unusually low gene turnover rate above the genomospecies level), or it is accessory (flexible) and is quickly gained or lost depending on the circumstances or the environmental pressure. Compared to *Pelagibacterales*, “*Ca.* Actinomarinales” have lower values of differential gene content across strains and a much smaller fraction of flexible genomic islands, with only a major one involved in cell envelope diversification. The latter probably responds to a strong selective pressure to evade phages (25), providing further evidence that phage population control applies to streamlined species like any other. However, the phenotypic diversity at the level of physiology might be drastically reduced compared to larger genomes. This fits well with a *K*-ecological strategy, characterized by steady population sizes that do not depend on hoarding resources as soon as they become available—as copiotrophic *r*-strategists do.

## MATERIALS AND METHODS

### Phylogenomic analysis.

All the available genomes belonging to the class *Acidimicrobiia* according to the GTDB (*Actinobacteria* based on the NCBI classification) as well as several reference genomes from nearby classes (*Actinobacteria*, *Coriobacteriia*, and *Themoleophilia*) were downloaded (accessed in January 2020). Additionally, SAGs classified as “*Ca*. Actinomarina” based on 16S rRNA gene phylogeny obtained in reference 17 were also included in the analysis. Genomes with a completeness of <50% and contamination of >5% based on CheckM (52) were removed from the analysis. Phylogenomic trees were built using phylophlan (53), and the resulting tree was analyzed using iTOL (54). A 16S rRNA gene phylogenetic tree was inferred using the neighbor-joining approach in MEGA7 (55) with 1,000 bootstraps and the Jukes-Cantor model of substitution.

### Pangenome analysis.

The comparison of encoded proteins among genomospecies can be affected by the absence of a certain protein due to the incompleteness of SAGs. Therefore, we combined several genomes from the same genomospecies to represent a “single” genome, i.e., pangenome. Only genomospecies with at least five genomes were considered. Proteins were clustered at 70% identity using cd-hit (global alignment) (56). This threshold is lower than the average nucleotide identity [ANI] among genomes from the same genomospecies. The pangenome of the class “*Ca.* Actinomarinales” was then analyzed using GET_HOMOLOGUES (57) considering a threshold of 30% identity and 75% alignment to consider ortholog proteins. The same approach was used for the subclade Ia.3 of the marine bacterium *Pelagibacterales*, following the genomospecies described in reference 18, namely, Ia.3/I, Ia.3V, Ia.3/VI, Ia.3/VII, and Ia.3/VIII were used. In this case, only five genomes (the largest and most complete) were used per group.

### Genome annotation and metabolism.

For each genome, coding DNA sequences from assembled contigs were predicted using Prodigal (58). tRNA and rRNA genes were predicted using tRNAscan-SE (59), ssu-align (60), and meta-rna (61). The inferred function was predicted comparing protein sequences against the NCBI NR database using DIAMOND (62) and against COG (63) and TIGFRAM (64) databases using HMMscan (65).

To reconstruct and understand the metabolic pathways present in the “*Ca.* Actinomarinales” genomes, proteins were aligned to the KEGG (Kyoto Encyclopedia of Genes and Genomes) using the BlastKoala tool (66). However, given that genomes are incomplete, for each genus we clustered all the proteins from the five most complete genomes at 70% identity using cd-hit (56). We added in the comparison two examples of streamlined genomes, the marine bacterium “*Candidatus* Pelagibacter” (strain HTCC7211 [GCA_000155895.1]) and the freshwater “*Ca.* Nanopelagicales” (strain IMCC25003 [GCA_002284855.1]), and the well-known copiotrophic bacterium E. coli (strain K-12) (GCA_000005845.2).

Both rhodopsin and heliorhodopsin were studied in more detail. Protein sequences were detected using HMMscan (65) against a custom HMM database containing thousands of sequences. Only hits with an E value of <1e^−15^ were considered. For each type, maximum likelihood phylogenetic trees with the closest relatives were performed using MEGA7 (55) with the following parameters: Jones-Taylor-Thornton model, gamma distribution with five discrete categories, and 100 bootstraps. Positions with less than 80% site coverage were eliminated. Before that, protein sequences were aligned with muscle (67).

### Retrieval of streamlined genomic parameters.

From each genus, we selected the most complete genomes to measure some genomic parameters. We also included in the analyses genomes of *Pelagibacterales*, “*Ca.* Nanopelagicales,” Prochlorococcus marinus, and E. coli. GC content was calculated using the gecee program from the EMBOSS package (68). The number of paralogs was retrieved using cd-hit, iterating from 90% to 30% in steps of 20% identity. The number of operons was measured using the webtool Operon-mapper (69). Intergenic spacers were calculated measuring the distance between consecutive genes.

### Genome comparisons.

Reciprocal BLASTN and TBLASTXs searches between genomes were conducted, leading to the identification of regions of similarity, insertions, and rearrangements. ANI between genomes was calculated using JSpecies software with default parameters (70). Intrapopulation sequence diversity within each group was calculated using the average nucleotide identity calculated by metagenomic reads (ANIr). Briefly, high-quality trimmed metagenomic reads (see “Metagenomic recruitment”) were recruited against reference genomes using BLASTN (71), with a cutoff 80% nucleotide identity and alignment length of ≥50 nucleotides.

### Genome reconstruction (composite genomes).

Composite genomes (CGs) of “*Ca*. Actinomarina” were reconstructed by coassembly of groups of SAGs. To minimize possible bias, the following were done. (i) Only groups of genomes with an ANI of >99% were used. (ii) These groups should have at least five representatives. (iii) All regions of the genome must be covered by at least two fragments. Figure S3 in the supplemental material shows the reconstruction of three composite genomes belonging to genomospecies G1.1, G2.1, and G2.3; in all the cases, the largest genome was used as a reference for assembly and rearranged to start at the *dnaA* gene. The high degree of synteny and completeness of the SAGs, the presence of contigs linking the beginning and the end of the genome, as well as the analysis of the GC skew give veracity to these genomes. Short of getting pure cultures, this is a good approach to reconstruct genomes of uncultivated microbes with realistic gene order and completion.

### Metagenomic recruitment.

To infer possible ecological distribution patterns, we used several metagenomic data sets (*Tara* Oceans [72] [BioProject accession number PRJEB1787] and GEOTRACES [BioProject accession number PRJNA385854] [21] expeditions as well as vertical profiles from the Western Mediterranean Sea [16] [BioProject accession number PRJNA352798], Red Sea [19] [BioProject accession number PRJNA289734], and North Pacific Ocean [BioProject accession number PRJNA352737] [20]) to recruit reads against “*Ca*. Actinomarina” genomes. The complete ribosomal operon gene cluster was manually removed from each genome sequence to avoid possible abundance bias (18). Only metagenomic reads that passed the quality criteria (Phred score ≥30, ≥50 bp long and with no ambiguous bases) filtered with Trimmomatic v0.36 (73) were aligned to the genomes using BLASTN (71). Metagenomic reads with a cutoff 98% nucleotide identity and alignment length of ≥50 nucleotides were used to compute the RPKG (reads recruited per kilobase of genome per gigabase of metagenome). A threshold of 5 RPKGs was established to consider the presence of a genome in a sample. For linear metagenomic representation, we used the same methodology but using a cutoff of 70% nucleotide identity over a minimum alignment length of 50 nucleotides. The alignments, together with the distribution of the reads according to the identity of the alignment (histogram) were plotted using the ggplot2 package in R.

### Evolutionary model analysis.

Starting from a preliminary set of 52 nearly complete genomes that represent all “*Ca.* Actinomarinales” genera but G5, we built orthologous gene clusters (OGC) with panX (74) and assessed genome completeness based on the presence of OGC for ribosomal proteins. For downstream analyses, we selected 23 genomes that harbor, as single copy, all the genes encoding a list of 30 nearly universal ribosomal proteins (75) plus 14 additional ribosomal proteins present in >85% of the genomes in the preliminary set. The selected genomes comprise 2,220 OGC, 306 of which represent strict single-copy core genes. Core-gene sequences were aligned with MAFFT (L-INS-i algorithm applied to translated sequences) (76) and columns with >90% of gaps were removed from the alignment. The core-gene sequence similarity tree was built by concatenating the alignments of the 306 core genes and running FastTree (options -gtr -gamma -mlacc 2 -slownni) (77), followed by RAxML (rapid hill-climbing algorithm, general tree reversible [GTR] model with gamma-distributed rates, FastTree tree provided as starting point) (78). The gene content tree was built with the phylogenomic reconstruction software Gloome (79) using four categories of gamma-distributed gain and loss rates and the core-gene sequence similarity tree as a guide.

To compare gene and genome evolution, we computed all pairwise distances among leaves in the sequence similarity and gene content trees. Then, we used nonlinear least-squares optimization to fit the observed relationship between both sets of distances to a model of gene and genome evolution subject to homologous recombination (see reference 28 for details).

### Data availability.

“*Ca*. Actinomarinales” composite genomes G1.1, G2.1, and G2.2 have been submitted to NCBI and are available under BioProject accession number PRJNA678693.
