# Supplementary material for: Genomes of the “Candidatus Actinomarinales” Order: Highly Streamlined Marine Epipelagic Actinobacteria
Source: mSystems. 2020 Dec 15;5(6):e01041-20. doi: 10.1128/mSystems.01041-20 (PMC7771536; doi:10.1128/mSystems.01041-20)
Supplement: FIG S1 [file mSystems.01041-20-sf001.pdf]

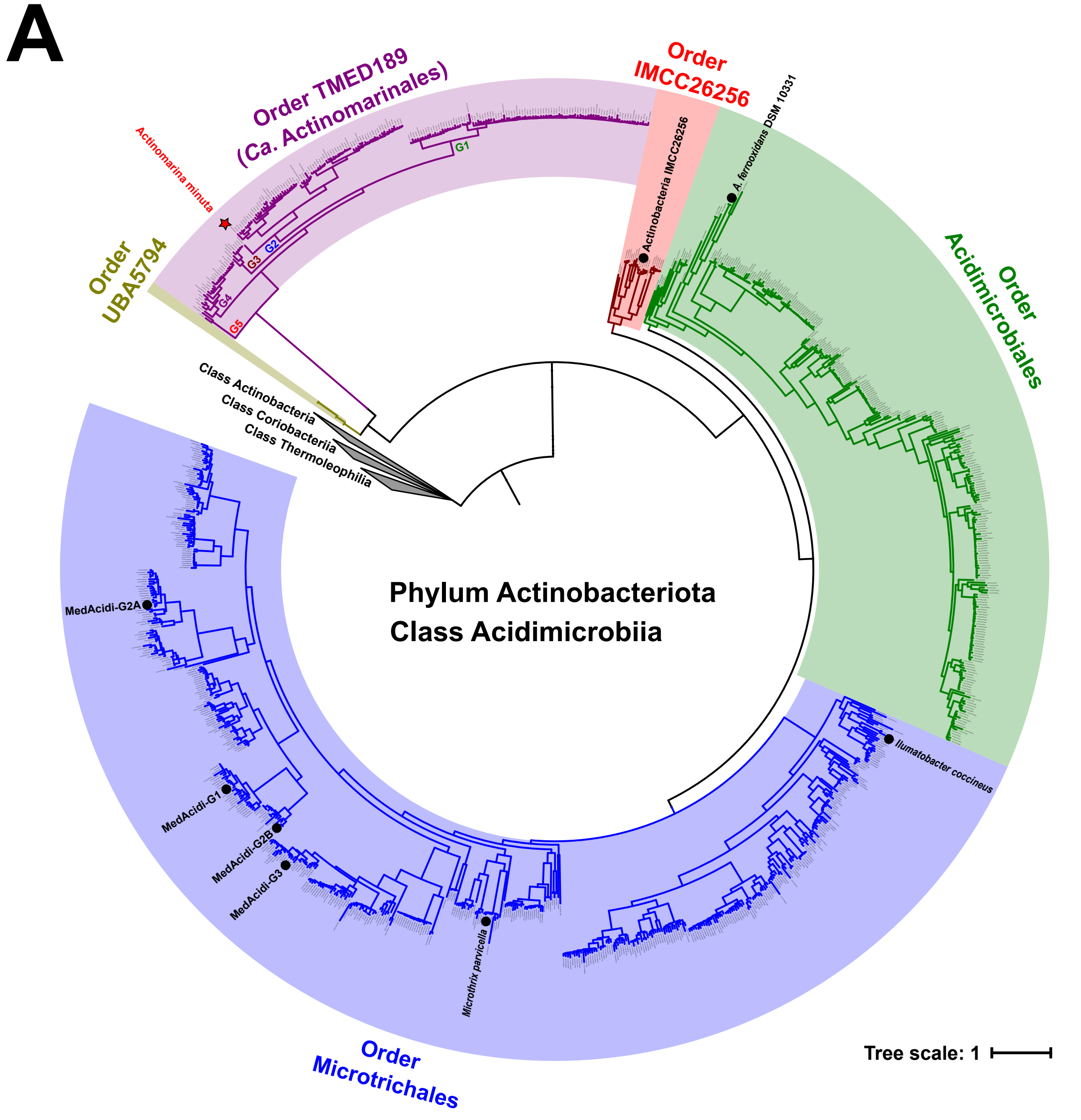

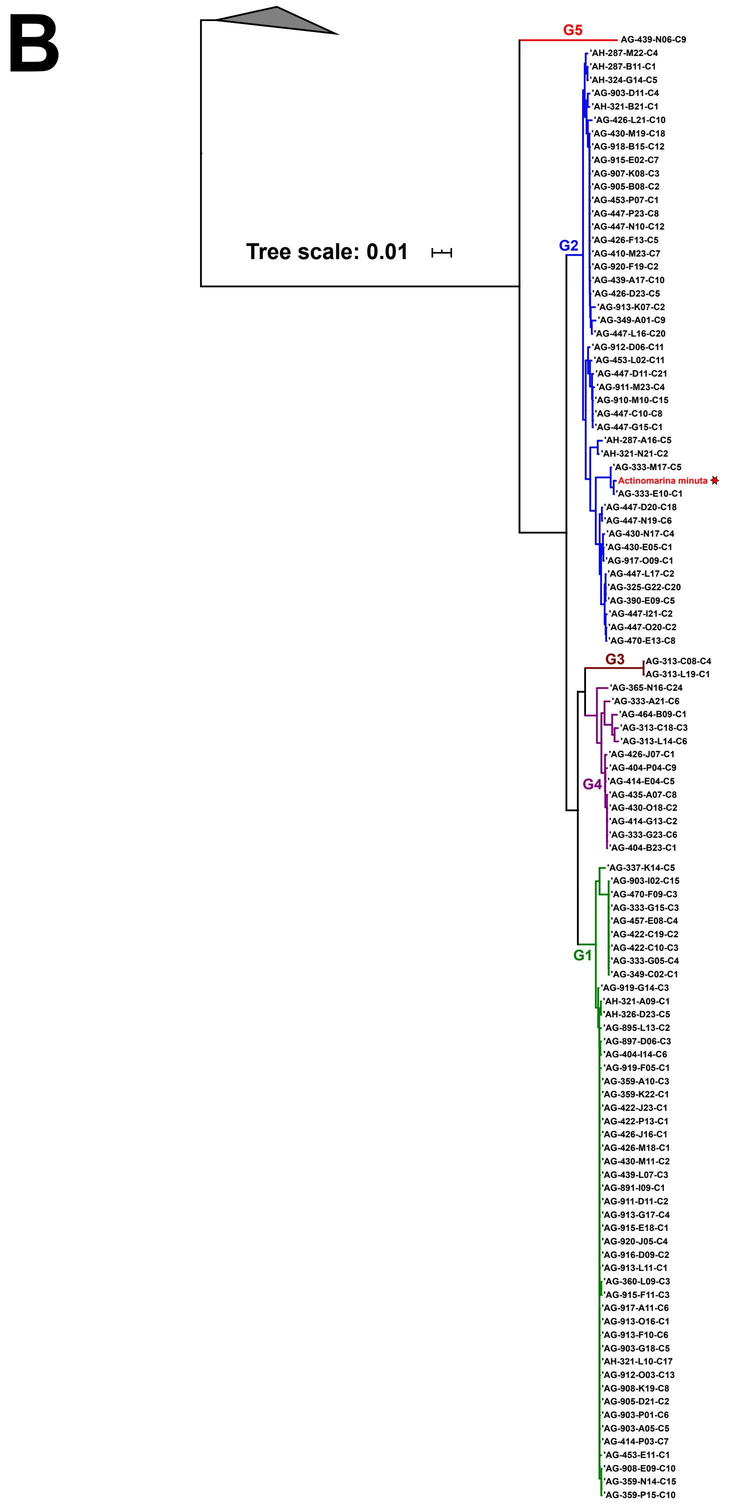

GTDB taxonomy

Phylum **Actinobacteriota**
Class **Acidimicrobiia**
Order **TMED189**
Family --
Genus --
Species--

NCBI taxonomy

Phylum **Actinobacteria**
Class **Actinobacteria**
Order ***Candidatus* Actinomarinales**
Family ***Candidatus* Actinomarinaceae**
Genus ***Candidatus* Actinomarina**
Species ***Candidatus* Actinomarina marina**
