# Supplementary figures and images for: Genomes of the “Candidatus Actinomarinales” Order: Highly Streamlined Marine Epipelagic Actinobacteria
Source: mSystems. 2020 Dec 15;5(6):e01041-20. doi: 10.1128/mSystems.01041-20 (PMC7771536; doi:10.1128/mSystems.01041-20)

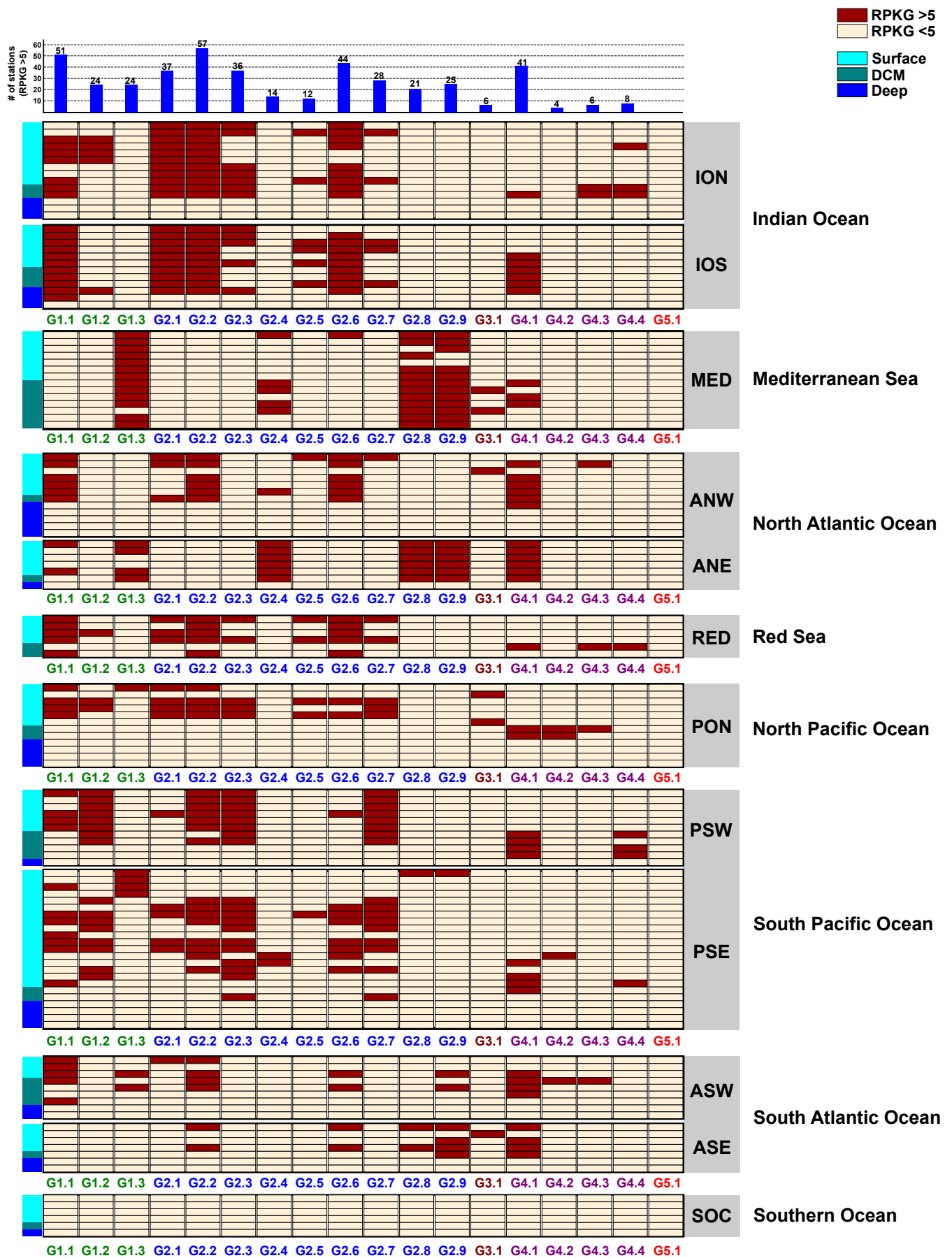

Supplement: FIG S2 [file mSystems.01041-20-sf002.pdf]

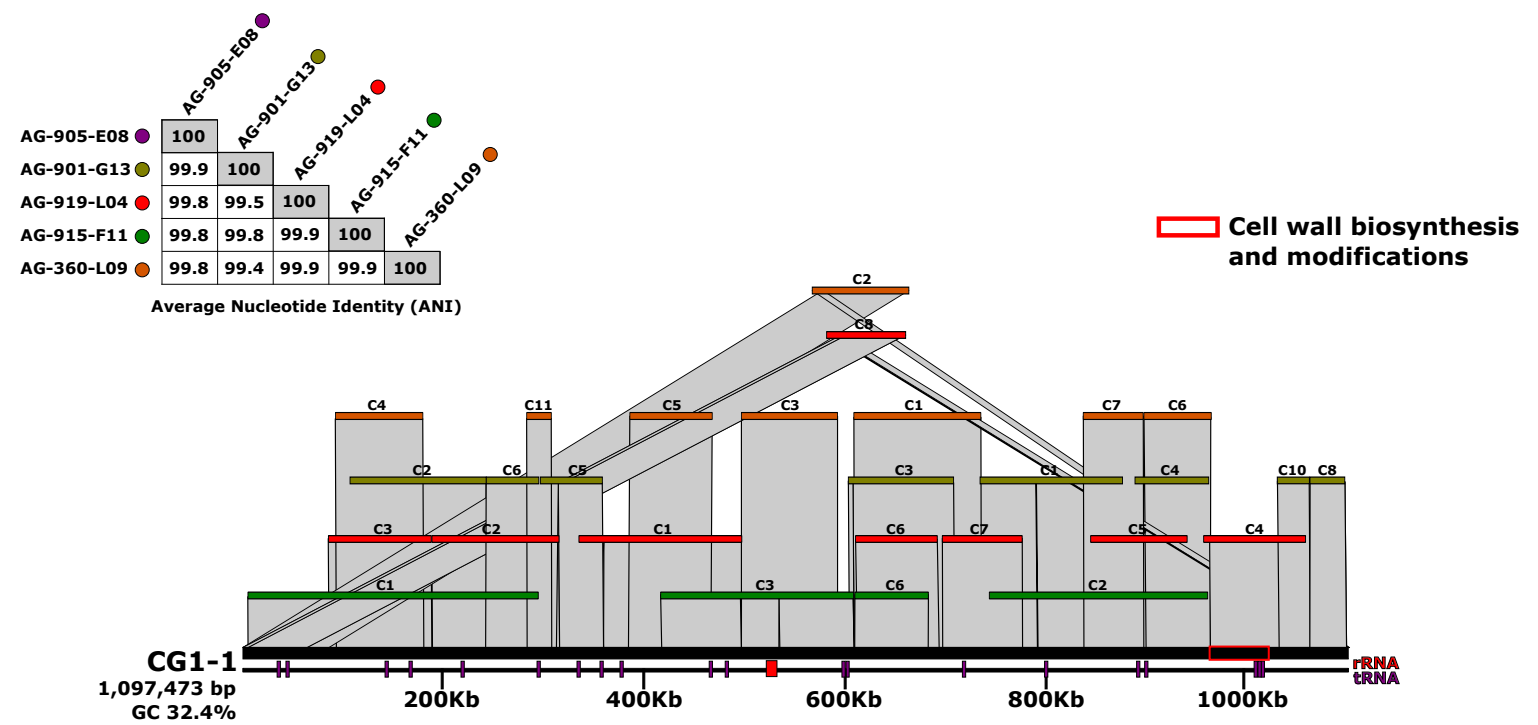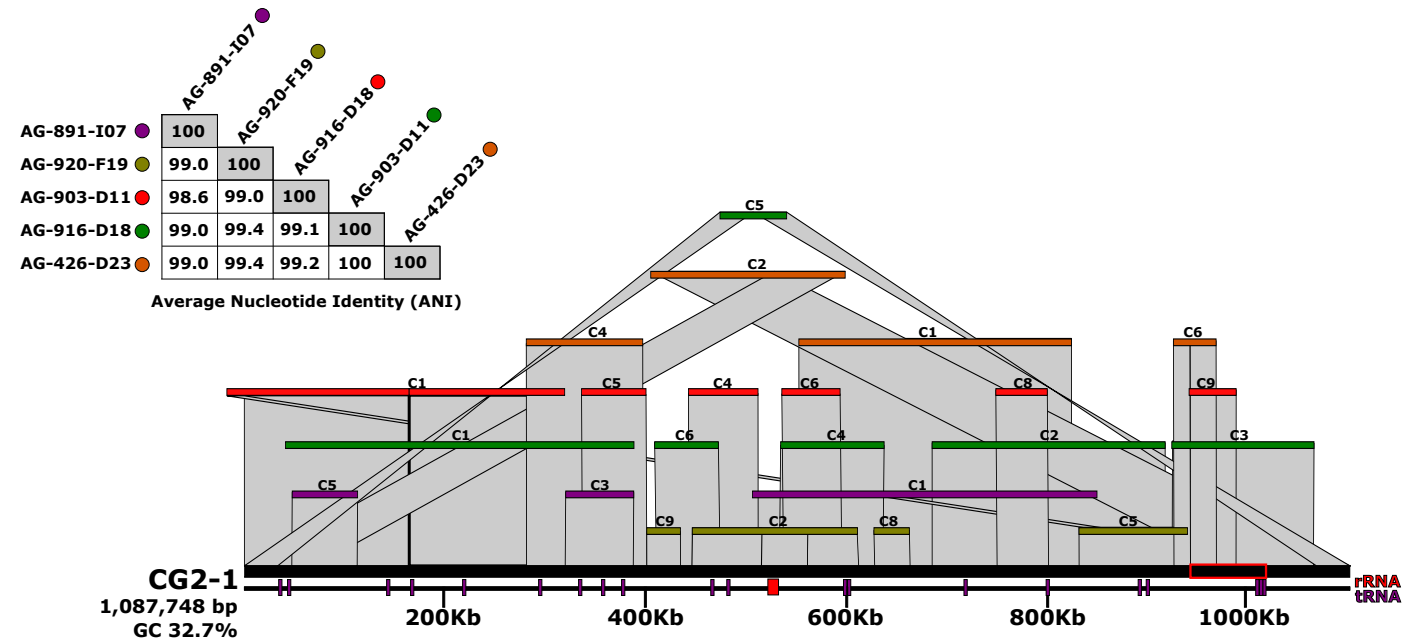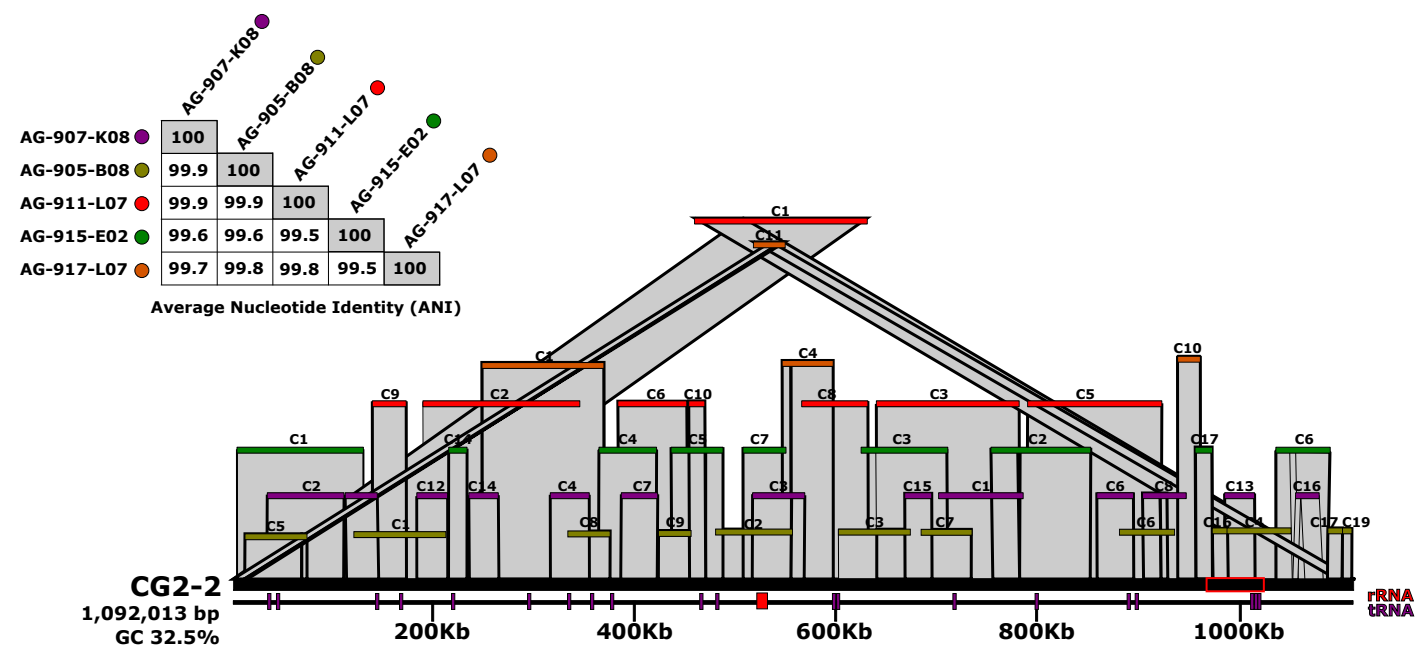

Supplement: FIG S3 [file mSystems.01041-20-sf003.pdf]

A

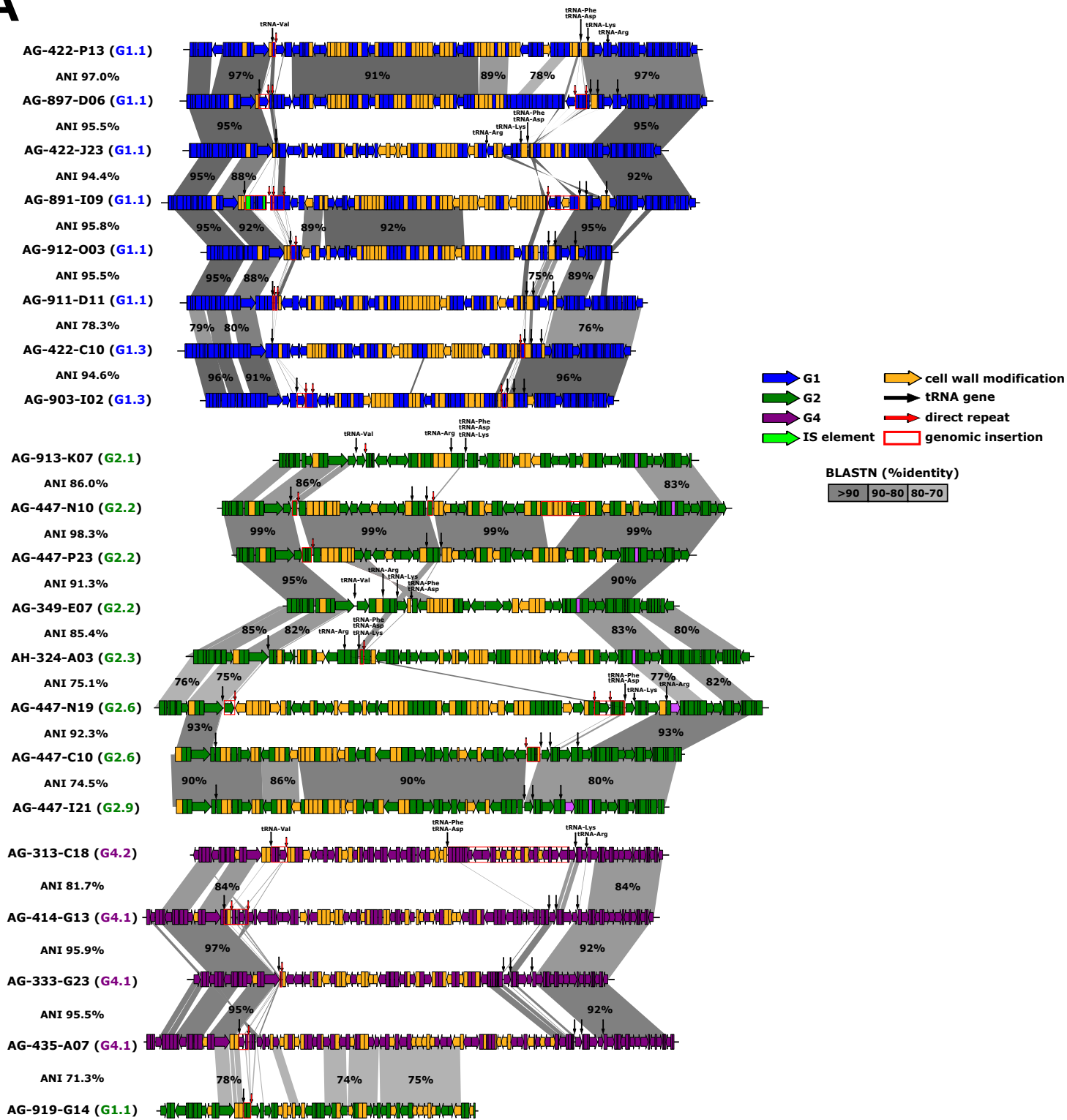

B

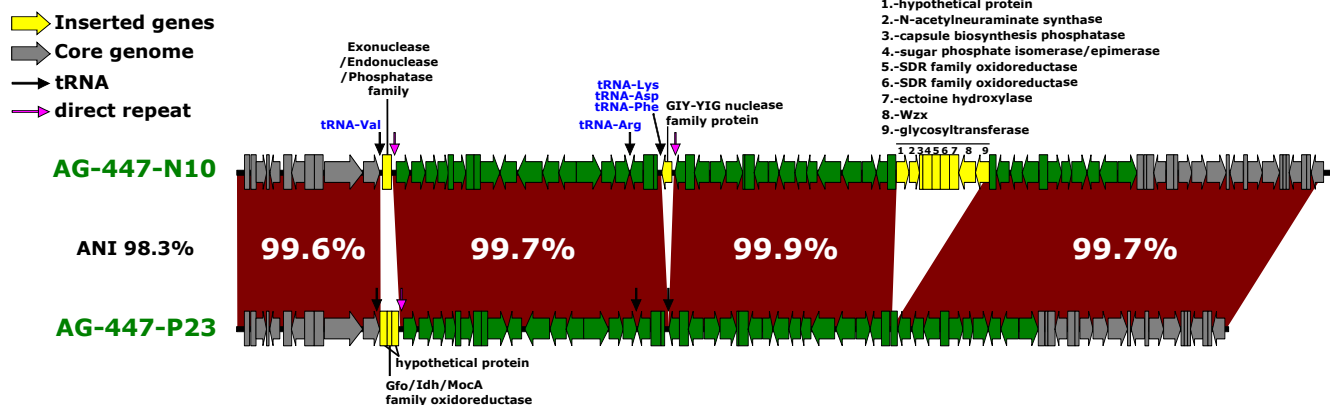

Supplement: FIG S4 [file mSystems.01041-20-sf004.pdf]

A

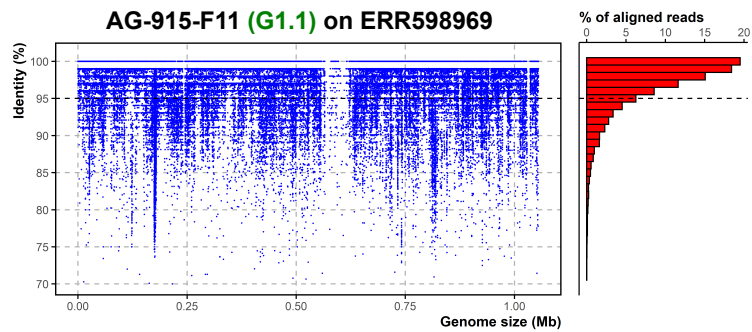

B

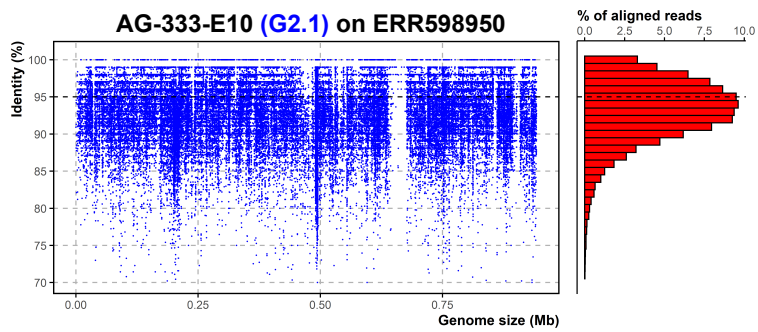

Supplement: FIG S5 [file mSystems.01041-20-sf005.pdf]

A

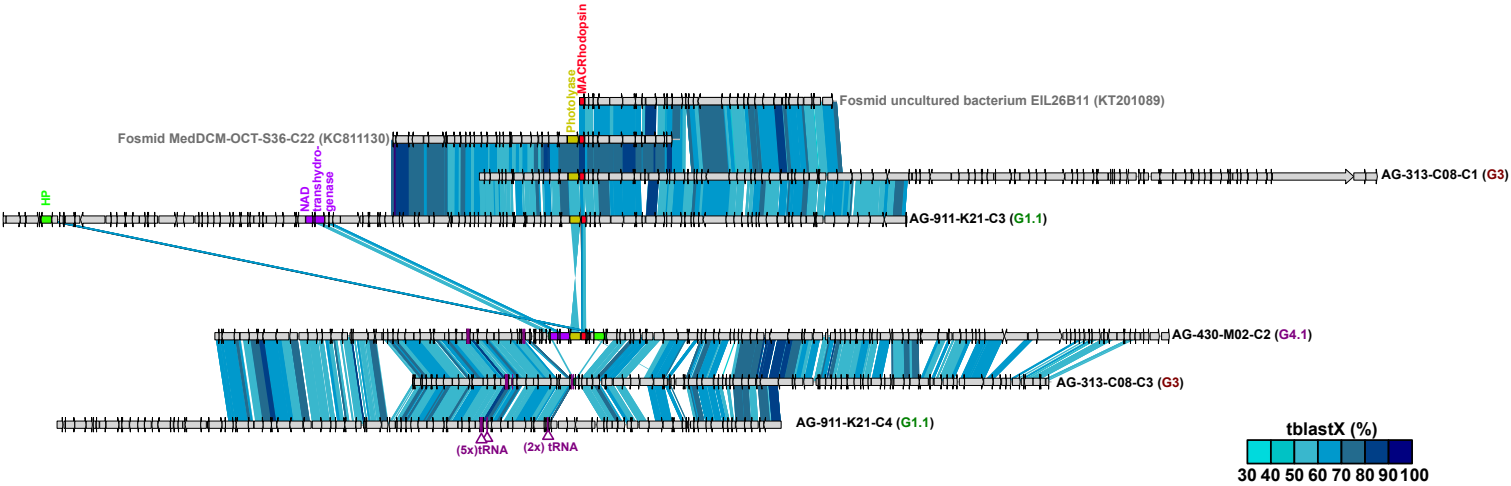

B

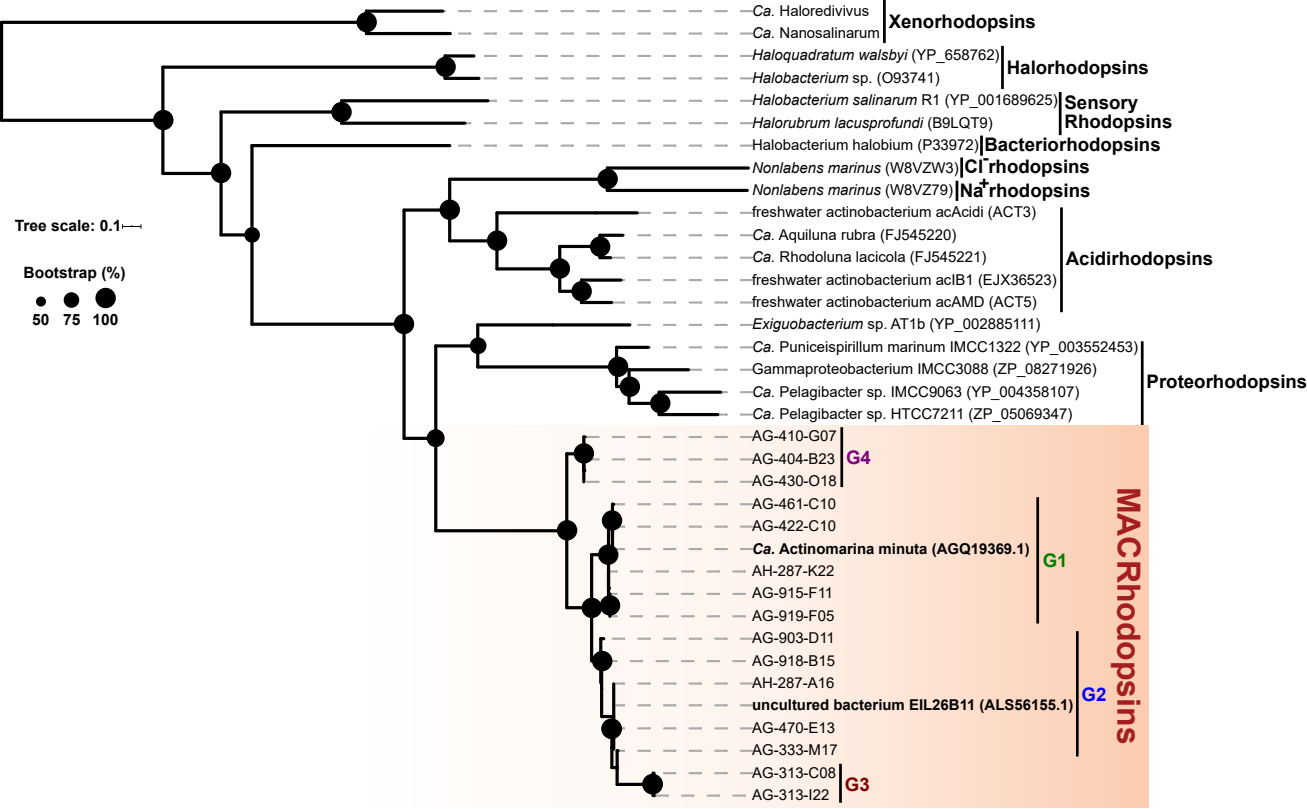

Supplement: FIG S6 [file mSystems.01041-20-sf006.pdf]

**A**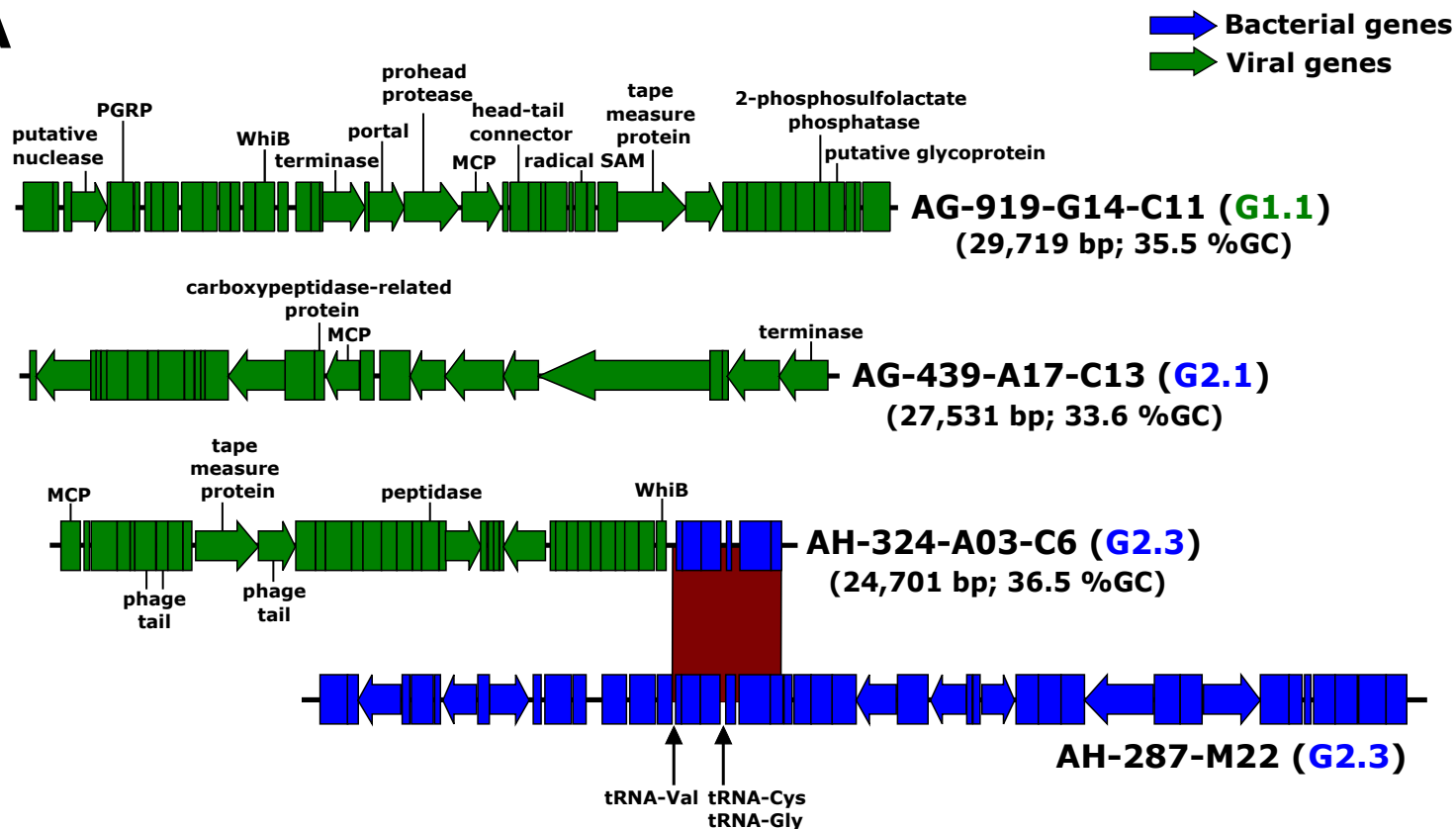**B**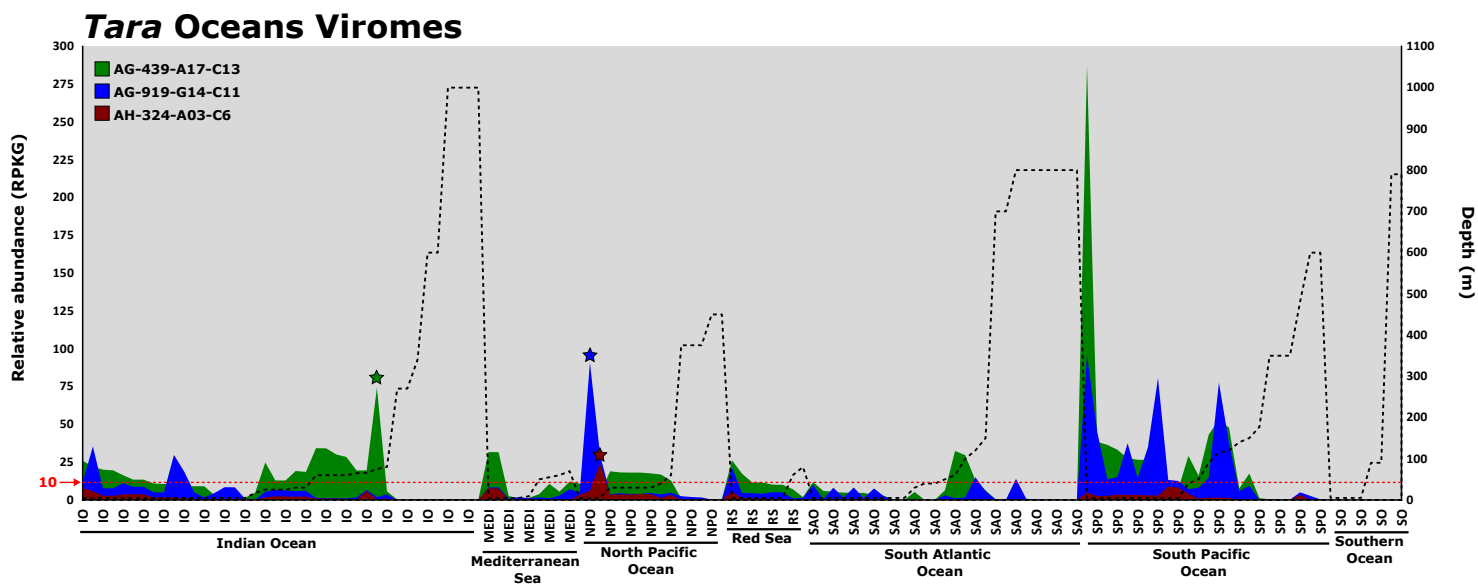**C**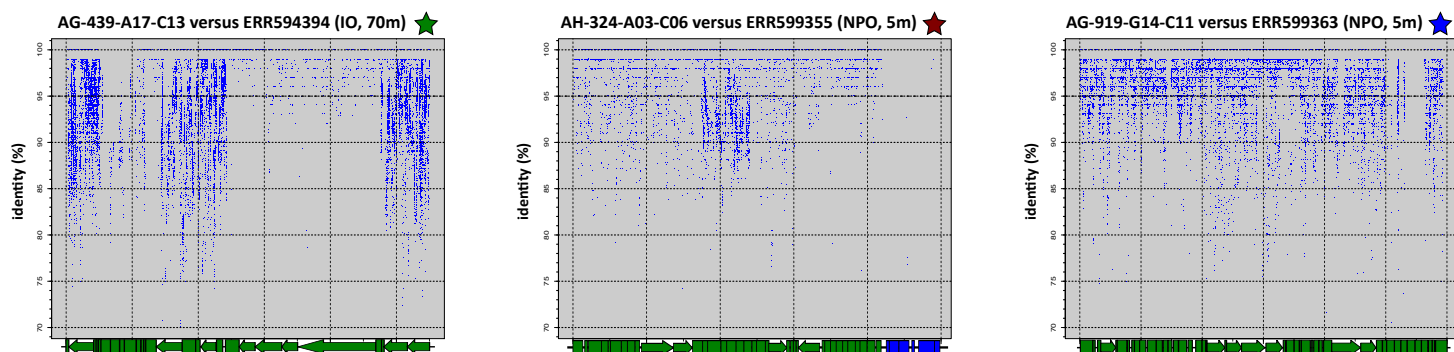

Supplement: FIG S7 [file mSystems.01041-20-sf007.pdf]
